# Autocrine IL-6 drives cell and extracellular matrix anisotropy in scar fibroblasts

**DOI:** 10.1016/j.matbio.2023.08.004

**Published:** 2023-11

**Authors:** Fiona N. Kenny, Stefania Marcotti, Deandra Belo De Freitas, Elena M. Drudi, Vivienne Leech, Rachel E. Bell, Jennifer Easton, María-del-Carmen Díaz-de-la-Loza, Roland Fleck, Leanne Allison, Christina Philippeos, Angelika Manhart, Tanya J. Shaw, Brian M. Stramer

**Affiliations:** a Randall Centre for Cell and Molecular Biophysics, King's College London, London, UK; b Centre for Inflammation Biology & Cancer Immunology, Department of Inflammation Biology, School of Immunology & Microbial Sciences, King's College London, London, UK; c Department of Mathematics, University College London, UK; d Centre for Ultrastructure Imaging, King's College London, UK; e Centre for Stem Cells and Regenerative Medicine, King's College London, London, UK; f Faculty of Mathematics, University of Vienna, Vienna, Austria

**Keywords:** Keloid, Fibrosis, Fibroblast, Scar, Extracellular matrix, IL-6

## Abstract

Fibrosis is associated with dramatic changes in extracellular matrix (ECM) architecture of unknown etiology. Here we exploit keloid scars as a paradigm to understand fibrotic ECM organization. We reveal that keloid patient fibroblasts uniquely produce a globally aligned ECM network in 2-D culture as observed in scar tissue. ECM anisotropy develops after rapid initiation of a fibroblast supracellular actin network, suggesting that cell alignment initiates ECM patterning. Keloid fibroblasts produce elevated levels of IL-6, and autocrine IL-6 production is both necessary and sufficient to induce cell and ECM alignment, as evidenced by ligand stimulation of normal dermal fibroblasts and treatment of keloid fibroblasts with the function blocking IL-6 receptor monoclonal antibody, tocilizumab. Downstream of IL-6, supracellular organization of keloid fibroblasts is controlled by activation of cell-cell adhesion. Adhesion formation inhibits contact-induced cellular overlap leading to nematic organization of cells and an alignment of focal adhesions. Keloid fibroblasts placed on isotropic ECM align the pre-existing matrix, suggesting that focal adhesion alignment leads to active anisotropic remodeling. These results show that IL-6-induced fibroblast cooperativity can control the development of a nematic ECM, highlighting both IL-6 signaling and cell-cell adhesions as potential therapeutic targets to inhibit this common feature of fibrosis.

## Highlights

• Patient-derived keloid scar fibroblasts consistently produce a globally aligned ECM network over millimeter length-scales in 2D culture.
• ECM anisotropy develops after rapid initiation of a supracellular actin network, which is mediated by activation of cell-cell adhesions.
• Intercellular coordination leads to monolayering of the fibrotic cells, which modeling suggests is sufficient to drive cell and subsequent ECM alignment.
• Keloid fibroblasts produce elevated levels IL-6, and autocrine IL-6 production is both necessary and sufficient to induce cell and ECM alignment.

## Introduction

Tissue fibrosis is a ubiquitous feature of numerous pathologies and responsible for up to 45% of worldwide deaths [1]. One pathology-agnostic change associated with fibrosis across organ systems is a reorganization of ECM architecture, leading to an aligned and bundled matrix, which is thought to alter the mechanical properties of the tissue. The development of a nematic ECM has primarily been studied in relation to cancers, which often develop fibrotic-like characteristics in the region surrounding the tumor [2,3]. Isolated cancer-associated fibroblasts (CAFs) are observed to produce an aligned cell derived matrix (CDM) in culture as a result of a variety of hypothesized autonomous CAF behaviors, such as enhanced contractility [4,5], integrin activation [4], increase in ECM components [6,7] and enhanced proteolytic remodeling of the ECM [8]. Additionally, increases in several upstream signaling factors, such as PDGF or TGF-β, have also been reported in CAFs to be involved in the alignment process [4,9]. However, it is currently unclear how these diverse CAF behaviors mechanistically lead to fibroblasts generating an aligned ECM over supracellular length scales, either *in vitro* or within fibrotic tissues.

Recent work in different CAF populations has suggested that a nematic ECM is driven by an initial patterning and alignment of the cells themselves. Park et al. [10] revealed that distinct contact inhibition of locomotion (CIL) dynamics in CAFs can induce a flocking-like behavior, which leads to a local alignment of fibroblasts, and it is this cellular pre-patterning that drives the long-range alignment of the underlying ECM. However, it is unknown whether this CIL-based mechanism is common to all CAF subtypes and if/how the previously mentioned CAF-specific behaviors are also playing a role (*e.g*., enhanced contractility, altered ECM components, TGF-β signaling).

The complexity of establishing whether there is a single CAF-based mechanism of ECM alignment is related to the fact that different cancers are highly heterogenous. Additionally, even the same cancer subtype is heterogenous in terms of the observed fibrosis-like characteristics, and histologically, different tumors display a spectrum of ECM alignment in the surrounding stroma [2]. Furthermore, different CAF isolates will show a range of alignment capacities in culture [10], which is likely dependent on a number of underlying variables that may be difficult to control for (e.g.*,* stage of cancer or levels of fibrotic activation).

While there have been numerous studies on CAF ECM alignment, there has been little exploration of fibroblast remodeling characteristics in more stereotypical fibrotic pathologies. Here we exploit keloid scars, which are a fibrotic skin pathology of unknown cause and lacking an animal model, to interrogate disease pathogenesis. Histologically, keloid tissue – similarly to many cancers – is observed to have a highly bundled organization of ECM fibers, which is in stark contrast to the basketweave appearance of normal dermis [11,12]. Keloids are a convenient pathology to study fibrotic mechanisms as they are histologically consistent, surgically removed at a similar stage of the condition (mostly when problematic and growing), and prevalent in the population. Additionally, it is also sometimes possible to obtain patient-matched control fibroblasts for experimentation. The consistency and prevalence of tissue makes it possible to precisely control experiments, which is arguably more difficult in most other fibrotic conditions.

Using a new quantitative algorithm to rapidly analyze the alignment of fibrillar features in biological images [13], we reveal that isolated patient-derived keloid fibroblasts – despite their long-term culture – consistently maintain a unique capacity to produce a highly aligned and bundled ECM. The mechanism of keloid ECM alignment is initiated by autocrine IL-6 production causing patterning and alignment of the fibroblasts themselves. Downstream of IL-6, keloid cells generate cell-cell adhesions that create a supracellular actin network leading to monolayering and alignment of the population. This underlying patterning of the keloid fibroblasts enables the cells to actively remodel and align an isotropic ECM. These data reveal that scar fibroblasts can cooperate in a supracellular fashion to actively remodel their environment to generate ECM anisotropy.

## Results

### Patient-derived scar fibroblasts maintain a unique capacity to align and generate an anisotropic ECM in culture

**Fig. 1:**
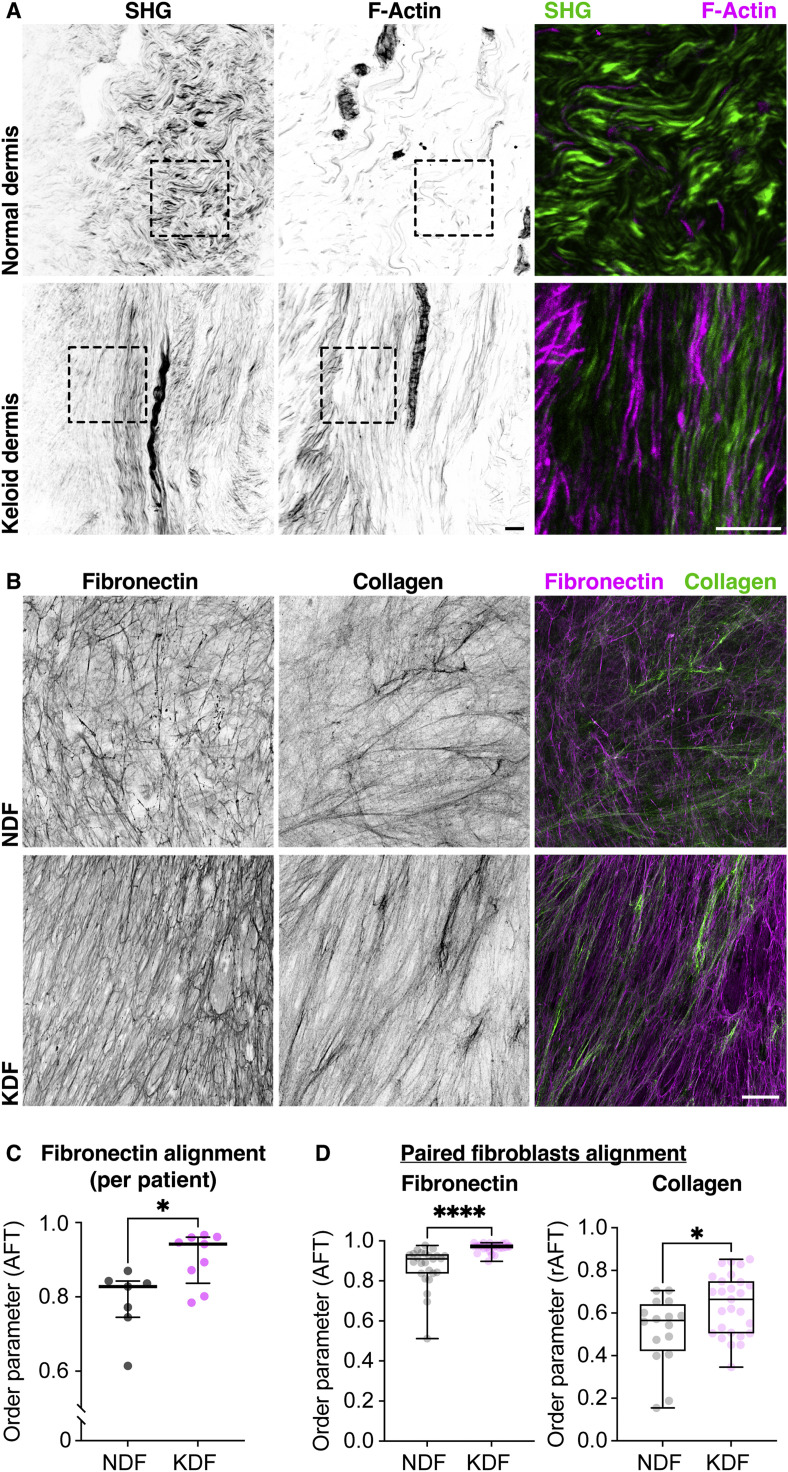
Keloid fibroblasts produce an aligned ECM both *in vivo* and *in vitro*. **(A)** Second Harmonic Generation (SHG) imaging and F-actin staining of keloid and normal dermis reveal highly aligned cells and ECM in keloid tissue. Note that there is very little F-actin in cells within the normal dermis. **(B)** Cell derived matrices (CDM) from isolated normal dermal fibroblasts (NDF) and keloid dermal fibroblasts (KDF) showing aligned fibronectin and collagen fibers from keloid cells. **(C)** Quantification of fibronectin alignment from individual patient samples highlights that KDF isolated from keloid tissues consistently produce an aligned ECM. *p = 0.0115, Mann-Whitney two-tailed test. Bars show medians and interquartile ranges for each patient sample; each datapoint is displayed as a dot (*n* = 7 NDF, 9 KDF biological replicates). **(D)** Quantification of ECM alignment in patient-matched KDF and NDF populations isolated from within the scar or the wound margin shows that the alignment phenotype is specific to the scar-associated fibroblasts. ****p < 0.0001, *p = 0.0267, Mann–Whitney two-tailed test. Boxplots show medians, 25th and 75th percentiles as box limits, minimum and maximum values as whiskers; each datapoint is displayed as a dot (*n* = 25 NDF, 27 KDF images for fibronectin, *n* = 16 NDF, 27 KDF for collagen, from one paired biological replicate). Scale bars, 20 µm.

Keloid scars are composed of a bundled ECM [12] with fibers showing increased alignment within the dermis (Fig. 1A and Supplemental Fig. 1A) [11,12,14]. Additionally, the orientation of the scar-associated fibroblast actin networks correlates with the direction of the surrounding ECM (Fig. 1A), highlighting a relationship between cell and ECM alignment. Due to frequent surgical removal of keloid scars [15], we were able to isolate primary keloid fibroblasts from numerous patient samples to examine their capacity to produce a cell derived matrix (CDM) in culture [16]. Analysis of ECM anisotropy using a high throughput image analysis algorithm, Alignment by Fourier Transform (AFT) [13,17] (Fig. 1B,C and Supplemental Fig. 1B,C), revealed that keloid patient fibroblasts (KDF) produced a highly aligned fibronectin and collagen matrix in culture compared to normal dermal fibroblasts (NDF). Scanning electron microscopy of CDMs also revealed a highly bundled and aligned ECM ultrastructure in keloid samples (Supplemental Fig. 1D). Interestingly, while it has been noted that fibroblast populations can lose some physiological differences upon culture [18,19], keloid cells maintained this unique capacity to generate an aligned ECM for numerous passages. This ECM alignment phenotype was consistent across patient samples revealing that this feature was strongly correlated with disease (Supplemental Fig. 1E). Additionally, a comparison of keloid fibroblasts with adjacent normal cells from the same patient demonstrated that the alignment phenotype was unique to fibroblasts within the scar tissue, showing that the behavior was specific to a particular cell state rather than a general fibroblast phenotype of the patient (Fig. 1D). Interestingly, fibroblasts isolated from normal scar tissue showed a similar capacity to produce an aligned ECM, suggesting that this behavior is a general feature of scar-associated fibroblasts (Supplemental Fig. 2A,B). These data reveal that isolated keloid fibroblasts consistently maintain a capacity to produce an anisotropic ECM in culture.

**Fig. 2:**
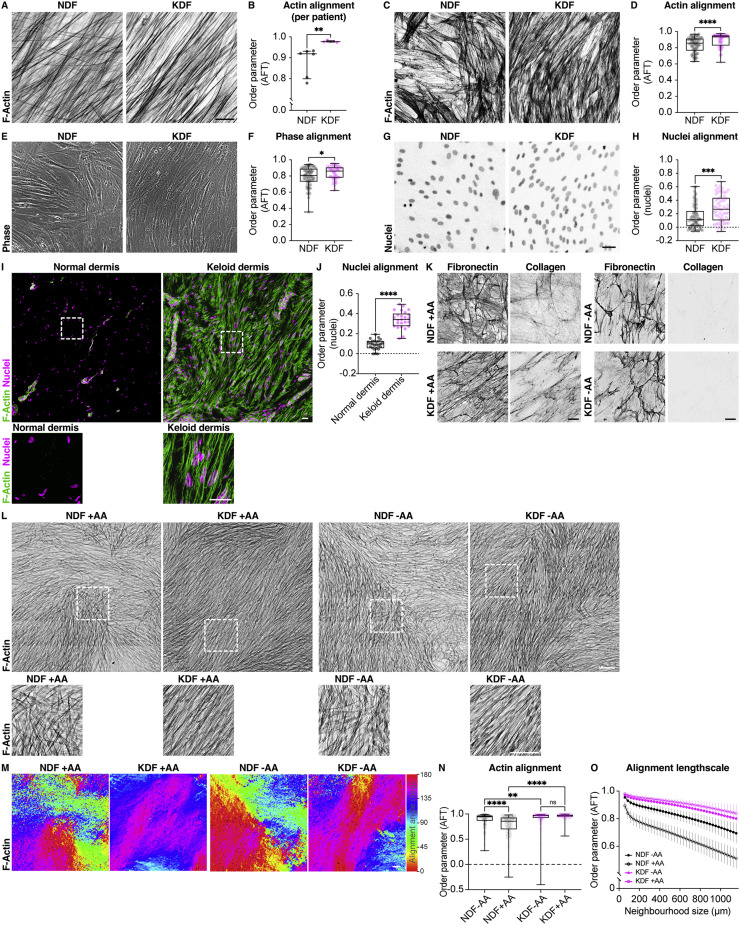
*In vitro* and *in vivo* alignment of keloid fibroblasts. (**A**) Normal dermal fibroblasts (NDF) and keloid dermal fibroblasts (KDF) cultured *in vitro* for 5 days and stained for F-actin reveal that KDF have an aligned actin network. Scale bar, 20 µm. **(B)** Quantification of actin alignment from individual patient samples cultured for 5 days highlights that KDF consistently align in culture. **p = 0.0043, Mann-Whitney two-tailed test. Bars show medians and interquartile ranges for each patient sample; each datapoint is displayed as a dot (*n* = 6 NDF, 5 KDF biological replicates). **(C,E,G)** NDF and KDF cultured *in vitro* for 24 h and imaged by fluorescence or phase microscopy [F-actin (C), phase (E), and nuclei (G)]. Scale bar, 50 µm. **(D,F,H)** Quantification of F-actin alignment (D), phase image alignment (F), or nuclear orientation alignment (H). Measures of alignment in phase images or nuclear orientation correlate with the increased F-actin alignment. ****p < 0.0001, ***p = 0.0002, *p = 0.0424, Mann–Whitney two-tailed test. Boxplots show medians, 25th and 75th percentiles as box limits, minimum and maximum values as whiskers; each datapoint is displayed as a dot (*n* = 67 NDF, 60 KDF images from 4 NDF and 3 KDF biological replicates). **(I)** F-actin and nuclear staining of normal dermis and keloid dermis shows that the increased actin fibers in keloid tissue appear aligned. Bottom panels are high magnification images of regions highlighted by hatched boxes. Scale bars, 20 µm. **(J)** Quantification of the orientation of the long axis of nuclei as a proxy for cell alignment in normal dermis and keloid dermis reveals that keloid cells are more highly aligned. ****p < 0.0001, Mann–Whitney two-tailed test. Boxplots show medians, 25th and 75th percentiles as box limits, minimum and maximum values as whiskers; each datapoint is displayed as a dot (*n* = 25 image neighborhoods from one biological replicate for each population). **(K)** NDF and KDF cultured in the presence (left) or absence (right) of ascorbic acid (AA) for 5 days. Decellularization and subsequent immunostaining for fibronectin or collagen reveals significantly reduced deposition of both ECM components in the absence of AA. Scale bar, 20 µm. **(L)** NDF and KDF cultured with or without ascorbic acid (AA) and imaged for F-actin. Bottom panels are high magnification images of regions highlighted by hatched boxes. Scale bar, 100 µm. **(M)** Heatmaps revealing the orientation of actin fibers in (L). Broad regions of coherence are visible in KDF compared to NDF, regardless of the presence of ascorbic acid (AA). Scale bar, 100 µm. **(N)** Quantification of actin alignment in (L) reveals that the presence of ascorbic acid (AA) – and an ECM – does not alter cell alignment in KDF. However, it significantly decreases alignment in NDF. ****p < 0.0001, **p = 0.0056, ns *p* = 0.2523, Kruskal-Wallis and Dunn's multiple comparisons test. Boxplots show medians, 25th and 75th percentiles as box limits, minimum and maximum values as whiskers; each datapoint is displayed as a dot (*n* = 100 randomly selected neighbourhoods from 3 NDF and 3 KDF biological replicates). **(O)** Quantification of actin and alignment over an increasing area in NDF and KDF cells with or without ascorbic acid (AA) reveals a faster decay for NDF in the presence of AA, suggesting a decrease in alignment over long length-scale.

We next examined whether fibroblast ECM alignment *in vitro* was correlated with alignment of the cells themselves by visualizing the actin network of confluent cultures and measuring actin network anisotropy. Comparing keloid, normal, and scar fibroblast alignment scores revealed that the actin network of keloid and normal scar cells was indeed more aligned in culture (Fig. 2A,B, Supplemental Fig. 2C–E). Additionally, compared to normal fibroblasts, keloid cells showed increased alignment in as little as 24 h and maintained these differences over time in culture (Fig. 2A–H). Additional measures of cell alignment correlated with the alignment of the actin network, such as anisotropy of phase contrast images and orientation of the major axis of nuclei, further confirming the significantly greater alignment of keloid versus normal dermal fibroblasts (Fig. 2E–H). Analysing actin networks and nuclear orientation in keloid tissues also revealed an increased alignment *in vivo* (Fig. 2I,J), similar to the increased alignment of the ECM (Supplemental Fig. 1A). Mathematical modeling has suggested that long-range alignment of fibroblasts is driven by feedback with the ECM [20]. We therefore cultured fibroblasts without the addition of ascorbic acid, which is essential for collagen processing and subsequent deposition in humans. The lack of ascorbic acid also significantly reduces fibronectin fiber formation as there is feedback between fibronectin deposition and collagen fibrillogenesis (Fig. 2K) [21,22]. However, the absence of ascorbic acid did not affect keloid cell alignment over short or long length-scales, counter to the hypothesis that fibroblast alignment requires feedback from the ECM (Fig. 2L–O) [20,23]. Interestingly, addition of ascorbic acid led to a further reduction in the alignment of normal fibroblasts revealing that the presence of ECM overrides an inherent capacity of non-fibrotic fibroblasts to order themselves in 2-D culture, which data suggest is related to the ordered packing of spindle shaped cells (Fig. 2L–O) [24]. These findings show that there are cell autonomous differences, independent of the ECM, that initiate keloid fibroblast actin network anisotropy.

### Autocrine IL-6 production in scar fibroblasts is both necessary and sufficient to drive cell and ECM anisotropy

**Fig. 3:**
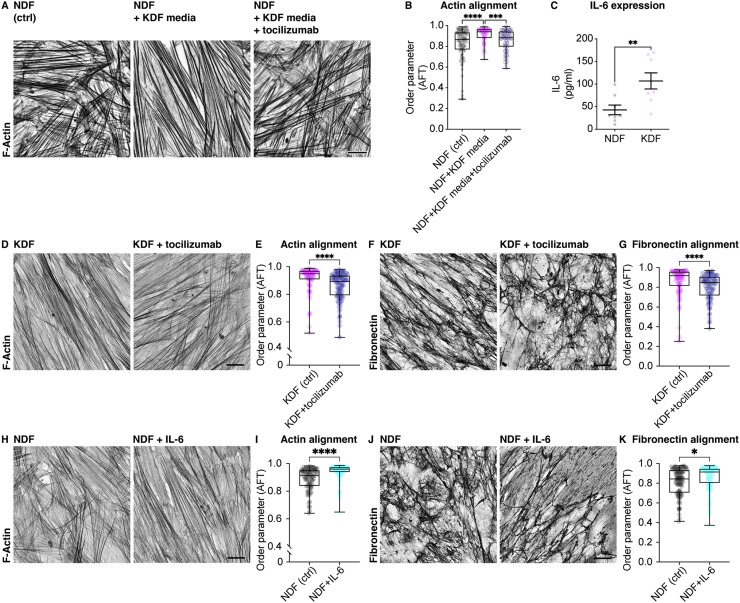
IL-6 is necessary and sufficient for keloid fibroblast cell and ECM alignment. **(A)** Normal dermal fibroblasts (NDF) treated with keloid dermal fibroblast (KDF) conditioned media or with KDF conditioned media plus tocilizumab and stained for F-actin. **(B)** Quantification of actin alignment in (A) reveals that KDF conditioned media increases NDF actin alignment, which is inhibited by the IL-6 inhibitor, tocilizumab. ****p < 0.0001, ***p = 0.0001, Kruskal-Wallis and Dunn's multiple comparisons test. Boxplots show medians, 25th and 75th percentiles as box limits, minimum and maximum values as whiskers; each datapoint is displayed as a dot (*n* = 90 NDF and NDF + KDF media + tocilizumab, 85 NDF + KDF media images from 3 biological replicates). **(C)** Quantification of IL-6 in conditioned media by enzyme-linked immunosorbent assay reveals an increase in KDF. **p = 0.007, Mann-Whitney two-tailed test. Bars show means and standard error of the means for each patient sample; each datapoint is displayed as a dot (*n* = 8 biological replicates). **(D)** KDF with and without tocilizumab treatment and stained for F-actin. **(E)** Quantification of actin alignment in (D) reveals that tocilizumab inhibits alignment of KDF. ****p < 0.0001, Mann-Whitney two-tailed test. Boxplots show medians, 25th and 75th percentiles as box limits, minimum and maximum values as whiskers; each datapoint is displayed as a dot (*n* = 81 KDF (ctrl), 100 KDF + tocilizumab images from 3 biological replicates). **(F)** CDM from KDF with and without tocilizumab and stained for fibronectin. **(G)** Quantification of fibronectin alignment in (F) reveals that tocilizumab inhibits CDM alignment of KDF. ****p < 0.0001, Mann-Whitney two-tailed test. Boxplots show medians, 25th and 75th percentiles as box limits, minimum and maximum values as whiskers; each datapoint is displayed as a dot (*n* = 88 KDF (ctrl), 85 KDF + tocilizumab images from 3 biological replicates). **(H)** NDF with and without IL-6 treatment and stained for F-actin. **(I)** Quantification of actin alignment in (H) reveals that IL-6 increases alignment of NDF. ****p < 0.0001, Mann-Whitney two-tailed test. Boxplots show medians, 25th and 75th percentiles as box limits, minimum and maximum values as whiskers; each datapoint is displayed as a dot (*n* = 78 NDF (ctrl), 84 NDF + IL-6 images from 3 biological replicates). **(J)** CDM from NDF with and without IL-6 treatment and stained for fibronectin. **(K)** Quantification of fibronectin alignment in (J) reveals that IL-6 increases CDM alignment of NDF. *p = 0.011, Mann-Whitney two-tailed test. Boxplots show medians, 25th and 75th percentiles as box limits, minimum and maximum values as whiskers; each datapoint is displayed as a dot (*n* = 89 NDF (ctrl), 93 NDF + IL-6 images from 3 biological replicates). Scale bars, 20 µm.

Treatment of normal fibroblasts with conditioned media from keloid cells revealed the presence of a secreted factor that increased their actin alignment (Fig. 3A,B). As TGF-β1-induced myofibroblast differentiation is often thought to be a driver of scar fibroblast behavior [25,26], and autocrine TGF-β production has been reported to be involved in CAF alignment of ECM [9], we speculated that keloid fibroblast alignment may be correlated with increased TGF-β signaling. However, isolated keloid fibroblasts showed no difference in myofibroblast differentiation as evidenced by western blotting for α-Smooth Muscle Actin in cultured cells (Supplemental Fig. 3A), which is consistent with recent work suggesting that myofibroblasts are uncorrelated with keloid pathogenesis [27]. Additionally, keloid fibroblasts showed no obvious difference in gel contraction assays, or the magnitude of individual cell traction forces (Supplemental Fig. 3C–E), despite a report that CAF alignment of ECM correlates with enhanced contractility [4,5]. Furthermore, treatment of normal fibroblasts with exogenous TGF-β1 failed to induce actin alignment (Supplemental Fig. 3B). These data suggest that a capacity for actin and ECM anisotropy is not simply a TGF-β-induced contractile myofibroblast response.

In contrast to TGF-β, the cytokine IL-6 has consistently been reported to be elevated in keloid scars [28], [29], [30], [31], [32], [33], [34], [35]. Indeed, isolated cultures of keloid fibroblasts show an increase in IL-6 secretion into the media (Fig. 3C), which led us to hypothesize that the enhanced alignment capacity of keloid conditioned media may be related to increased IL-6. To examine whether autocrine IL-6 production in keloid cells was initiating cell and ECM alignment, we treated normal and keloid cells with the IL-6 receptor blocking antibody, tocilizumab. This revealed that inhibiting IL-6 signaling reduced both actin alignment and ECM anisotropy in keloid cells to levels observed in normal fibroblasts (Fig. 3D–G). Additionally, combining keloid fibroblast conditioned media with tocilizumab inhibited its capacity to induce alignment of normal cells (Fig. 3A,B). To examine whether IL-6 is also sufficient to increase the alignment process, normal fibroblasts were treated with exogenous IL-6, which led to an increase in both actin and ECM alignment to levels approaching what was observed in keloid fibroblasts (Fig. 3H–K). These data reveal that autocrine IL-6 production in keloid cells is both necessary and sufficient to induce ECM anisotropy.

### Scar fibroblasts generate a supracellular actin network and long-range alignment of the ECM

**Fig. 4:**
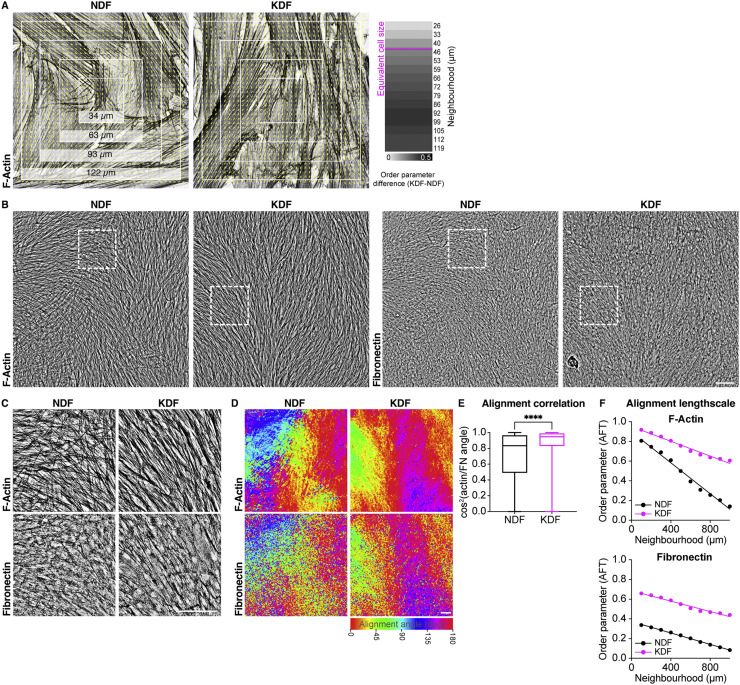
Keloid fibroblasts autonomously align supracellularly *in vitro*. **(A)** Quantification of actin alignment over an increasing area and subtracting the NDF alignment score from KDF reveals an optimal actin alignment difference within a 93 µm x 93 µm area. As an average KDF or NDF is approximately 44 µm x 44 µm in area, this optimal alignment area represents ∼3–4 cell diameters. **(B)** Normal dermal fibroblasts (NDF) and keloid dermal fibroblasts (KDF) cultured *in vitro*, stained for F-actin and fibronectin, and imaged over a millimeter area. **(C)** High magnification images of regions highlighted by hatched boxes in (B). **(D)** Heatmaps revealing the orientation of actin and fibronectin fibers in (B), showing broad regions of coherence in KDF. **(E)** Comparing the local orientation of actin and fibronectin fibers shows that KDF are more highly correlated with the ECM. ****p < 0.0001, Mann–Whitney two-tailed test. Boxplots show medians, 25th and 75th percentiles as box limits, minimum and maximum values as whiskers (*n* = 6241 local alignment vectors from one biological replicate for each population). **(F)** Quantification of actin and fibronectin alignment over an increasing area in NDF and KDF cells isolated from the same patient reveals an increased alignment over millimeter lengthscale. Scale bars, 100 µm.

The difference in actin alignment between normal and fibrotic cell types was not observed in single isolated cells (Supplemental Fig. 3F,G). However, an analysis of actin alignment over increasing neighborhood sizes revealed that alignment emerged in confluent cultures over an area that encompassed 3–4 cell diameters, showing that actin alignment was supracellular (Fig. 4A). We directly compared the actin network and ECM alignment in cell cultures, which revealed that actin and CDM orientation were more strongly correlated in keloid fibroblasts (Fig. 4B–E). We subsequently examined the length scale of this alignment. Analysing the decay in alignment scores over increasing distances revealed that the actin network and ECM from keloid fibroblasts remained significantly aligned over millimeter length scale (Fig. 4F). As long-range cellular alignment can be driven by shape anisotropy and cellular packing [24], and an enhanced cell aspect ratio is hypothesized to drive ECM alignment in CAFs [36], we wondered whether a distinct shape of keloid cells was driving their alignment. However, analysis of cell shape in individual cells or cells within a confluent culture revealed no difference in the aspect ratio or cell area between keloid and normal fibroblasts (Supplemental Fig. 3H–L). These data suggest that long-range actin and ECM alignment in keloid fibroblasts is correlated with the development of a supracellular anisotropic actin network.

### Keloid fibroblasts and IL-6 treated normal fibroblasts require cell-cell adhesions for supracellular alignment

We next examined keloid behaviors that may be downstream of IL-6 in controlling the cellular alignment process. One recently reported mechanism responsible for CAF-mediated ECM alignment was a differential capacity to undergo CIL, which leads to a migration-driven alignment of the cells; mathematical modeling revealed that CAF collision guidance can lead to cellular alignment and subsequent templating of an aligned ECM [10]. However, examination of keloid and normal fibroblast CIL dynamics in sparse cultures revealed no signs of collision guidance that could lead to reorientation of collisions into either an aligned or anti-aligned configuration [10]. Furthermore, there was no discernible difference between keloid and normal fibroblast reorientation after collision (Supplemental Fig. 3 M,N). These data reveal that while keloid fibroblasts show a similar capacity for producing an aligned ECM, the mechanism responsible appears unique compared to the collision guidance mechanism observed in CAFs.

**Fig. 5:**
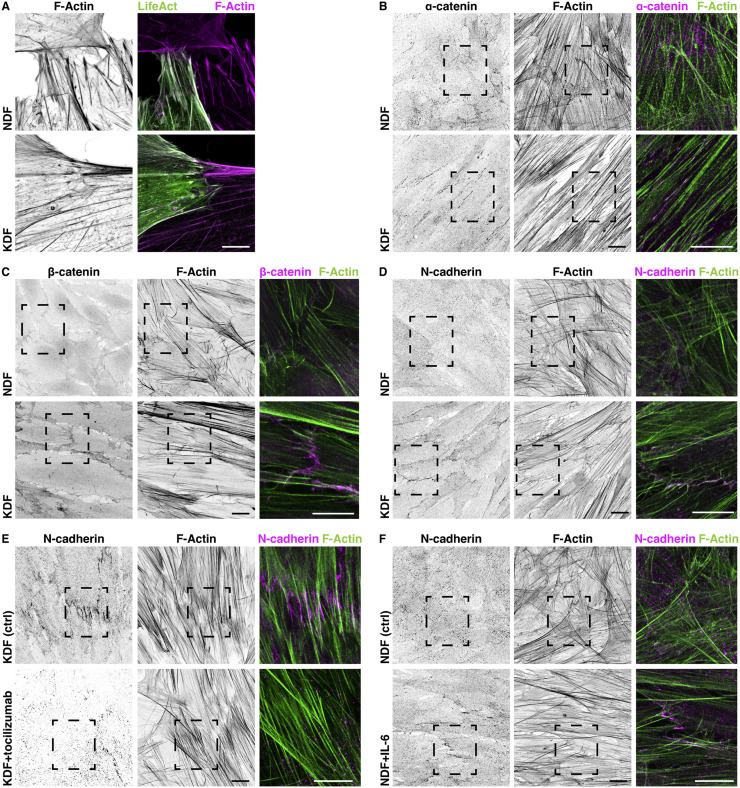
Keloid fibroblasts generate intercellular adhesions and monolayers in culture. **(A)** Staining normal dermal fibroblasts (NDF) and keloid dermal fibroblasts (KDF) for F-actin and mosaically labeling actin in cells by LifeAct transfection reveals supracellular actin fibers interconnecting KDF. Scale bar, 10 µm. **(B)** NDF and KDF stained for F-actin and α-catenin reveal enhanced localization of α-catenin at cell-cell junctions in KDF. Scale bars, 20 µm. **(C)** NDF and KDF stained for F-actin and β-catenin reveal enhanced localization of β-catenin at cell-cell junctions in KDF. Scale bars, 20 µm. **(D)** NDF and KDF stained for F-actin and N-cadherin reveal enhanced localization of N-cadherin at cell-cell junctions in KDF. Scale bars, 20 µm. **(E)** KDF control and tocilizumab-treated cells stained for F-actin and N-cadherin reveal a reduction in localization of N-cadherin at cell-cell junctions. Scale bars, 20 µm. **(F)** NDF control and IL-6-treated cells stained for F-actin and N-cadherin reveal an increase in localization of N-cadherin at cell-cell junctions. Scale bars, 20 µm. Right panels are high magnification images of regions highlighted by hatched boxes.

As keloid fibroblast actin fibers spanned the length of numerous cells (Fig. 4A) and mosaic labeling of fibroblasts revealed actin fibers precisely adjoining at cell boundaries (Fig. 5A), we wondered if intercellular coordination was driving the alignment process. Immunostaining for markers of cell-cell adhesions, including α-catenin, β-catenin, and N-cadherin (Fig. 5B–D), revealed that keloid fibroblasts developed prominent cell-cell junctions. While normal fibroblasts showed diffuse staining for these components, keloid cells showed punctate staining at regions of intercellular contacts. Furthermore, treatment of keloid fibroblasts with the IL-6 receptor inhibitor, tocilizumab, decreased N-cadherin puncta (Fig. 5E), while IL-6 treatment of normal fibroblasts increased puncta (Fig. 5F), indicating that intercellular adhesion development is downstream of IL-6 signaling.

**Fig. 6:**
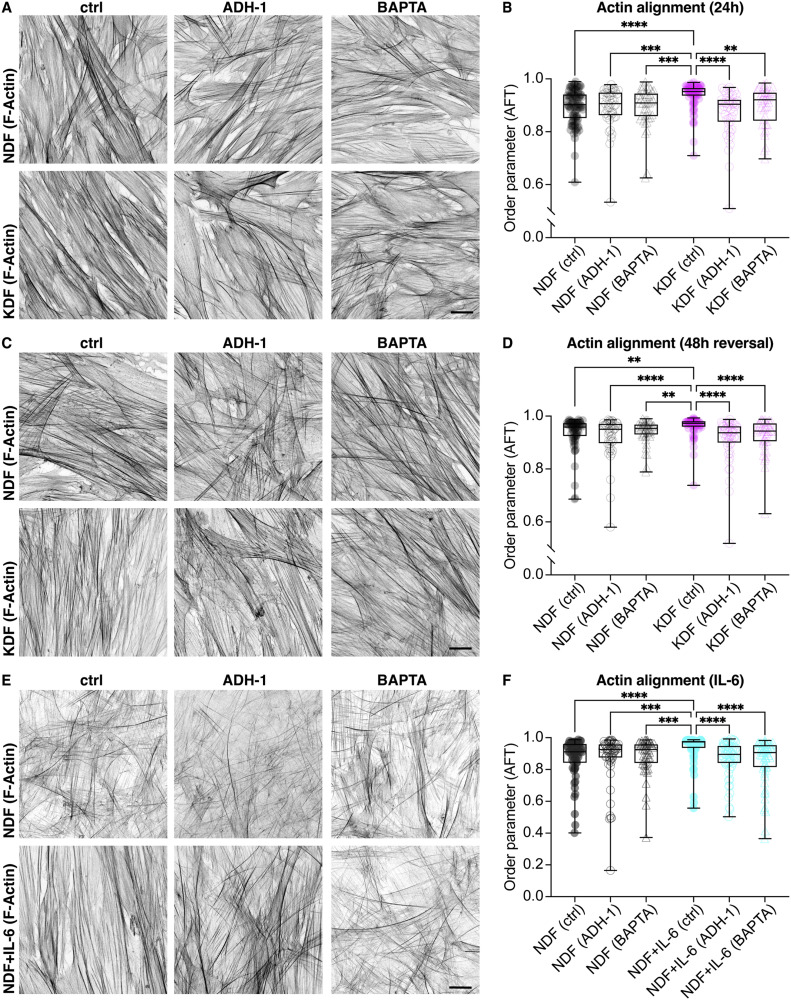
Inhibiting intercellular adhesions can prevent KDF alignment and IL-6 dependent alignment of NDF. **(A)** Normal dermal fibroblasts (NDF) and keloid dermal fibroblasts (KDF) treated immediately upon plating with the calcium chelator, BAPTA, or the N-cadherin inhibitor, ADH-1, cultured for 24 h, and stained for F-actin. **(B)** Quantification of actin alignment in (A) reveals a decrease in KDF alignment after inhibition of cell-cell adhesions. ****p < 0.0001; ***p = 0.0001 (KDF ctrl vs NDF ADH-1), ***p = 0.0003 (KDF ctrl vs NDF BAPTA); **p = 0.001, Kruskal-Wallis and Dunn's multiple comparisons test. Boxplots show medians, 25th and 75th percentiles as box limits, minimum and maximum values as whiskers; each datapoint is displayed as a dot (*n* = 47 [NDF (ctrl)], 46 [NDF (ADH-1)], 42 [NDF (BAPTA)], 45 [KDF (ctrl)], 47 [KDF (ADH-1)], 42 [KDF (BAPTA)] images from 5 NDF and 3 KDF biological replicates). **(C)** NDF and KDF cultured for 24 h, which is a sufficient for cell alignment (Fig. 2C–H), and subsequently treated with BAPTA or ADH-1 for 48 h before staining for F-actin. **(D)** Quantification of actin alignment in (C) reveals that inhibiting cell-cell adhesions leads to a reversal of actin network alignment in KDF. ****p < 0.0001, **p = 0.0028 (KDF ctrl vs NDF ctrl), **p = 0.002 (KDF ctrl vs NDF BAPTA), Kruskal-Wallis and Dunn's multiple comparisons test. Boxplots show medians, 25th and 75th percentiles as box limits, minimum and maximum values as whiskers; each datapoint is displayed as a dot (*n* = 54 [NDF (ctrl)], 48 [NDF (ADH-1)], [48 NDF (BAPTA)], 52 [KDF (ctrl)], 53 [KDF (ADH-1)], 47 [KDF (BAPTA)] images from 4 NDF and 4 KDF biological replicates). **(E)** NDF with and without IL-6 treatment plus inhibitors of cell-cell adhesions (BAPTA and ADH-1), stained for F-actin. **(F)** Quantification of actin alignment in (E) reveals that inhibition of cell-cell adhesions prevents IL-6-mediated alignment of NDF. ****p < 0.0001; ***p = 0.005 (NDF ADH vs NDF + IL-6 ctrl), ***p = 0.008 (NDF BAPTA vs NDF + IL-6 ctrl), ns *p* > 0.9999, Kruskal-Wallis and Dunn's multiple comparisons test. Boxplots show medians, 25th and 75th percentiles as box limits, minimum and maximum values as whiskers; each datapoint is displayed as a dot (*n* = 60 [NDF (ctrl), NDF (ADH1), NDF + IL-6 (ctrl), NDF + IL-6 (ADH1), NDF + IL-6 (BAPTA)], 51 [NDF (BAPTA)] images from 3 biological replicates). Scale bars, 20 µm.

As the data demonstrate a correlation between intercellular coordination and cell and ECM alignment, we examined actin network alignment after perturbing cell-cell adhesions by chelating calcium from the media with BAPTA [37] or blocking N-cadherin adhesions directly with an inhibitory peptide, ADH-1 [38]. Adding these inhibitors to the media upon plating fibroblasts was sufficient to inhibit actin network alignment within 24 h in culture (Fig. 6A,B). We also examined whether keloid fibroblast alignment could be reversed; adding cell-cell adhesion inhibitors after first allowing the keloid fibroblasts to align over 24 h in culture also decreased the alignment to control levels (Fig. 6C,D). Additionally, blocking cell-cell adhesions was sufficient to inhibit the IL-6-induced alignment of normal fibroblasts (Fig. 6E,F). These data reveal that cell-cell adhesions are essential for initiation and subsequent maintenance of supracellular alignment of keloid cells.

### Cell-cell adhesion formation in fibroblasts inhibits cellular overlap and aligns focal adhesions

**Fig. 7:**
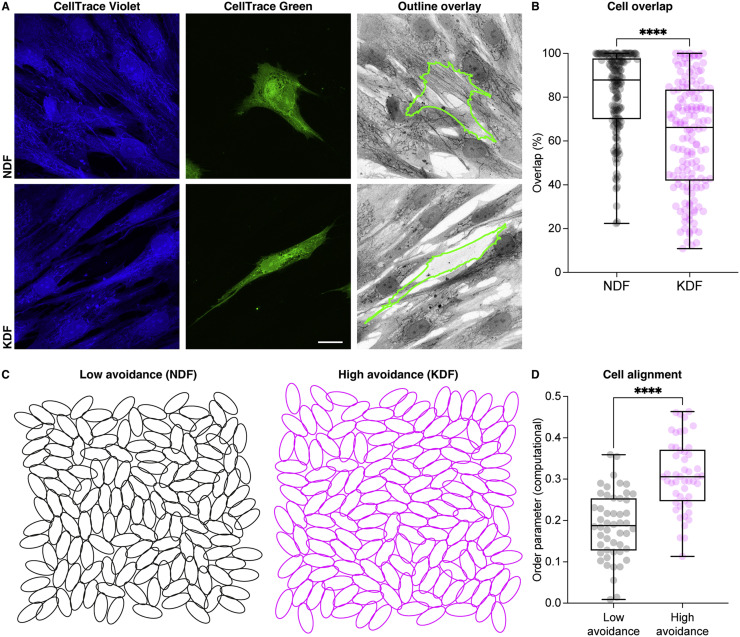
Keloid fibroblasts organize into a monolayer. **(A).** Normal dermal fibroblasts (NDF) and keloid dermal fibroblasts (KDF) mosaically labelled to examine the capacity of cells to overlap with neighbors. Scale bar, 20 µm. **(B)** Quantification of the area of overlap of the mosaically labelled cells in (A) with neighbors reveals that KDF shows reduced layering capacity. ****p < 0.0001, Mann–Whitney two-tailed test. Boxplots show medians, 25th and 75th percentiles as box limits, minimum and maximum values as whiskers; each datapoint is displayed as a dot (*n* = 154 NDF, 150 KDF images from 3 biological replicates for each population). **(C)** Example computational simulation results for two different degrees of cell-cell overlap avoidance (see Appendix): low avoidance (*e.g*., NDF), high avoidance (*e.g*., KDF). Overlap avoidance values were chosen to match migratory persistence as measured experimentally. **(D)** Quantification of the order parameter for the simulation results, revealing that an increase in cell-cell avoidance is sufficient to cause an increase in alignment scores. ****p < 0.0001, Mann–Whitney two-tailed test. Boxplots show medians, 25th and 75th percentiles as box limits, minimum and maximum values as whiskers; each datapoint is displayed as a dot (*n* = 50 NDF and KDF simulations).

We subsequently wanted to examine the consequences of the intercellular adhesions in the fibroblast population that allow for enhanced cell and ECM alignment. Keloid fibroblasts showed a reduced proliferative capacity at high cell density, suggesting that cell-cell adhesions may be leading to contact inhibition of proliferation (Supplemental Fig. 4A). Furthermore, live imaging of cell migration in confluent cultures revealed that keloid fibroblasts, unlike normal cells, were inhibited from migrating over neighboring cells leading to reduced migratory persistence (Supplemental Fig. 4B, D and Video 1). This suggests an alteration in CIL observed only upon cells reaching confluence. To examine this further, we mosaically labelled confluent cultures of fibroblasts to examine the degree of overlap between cells. Compared to normal fibroblasts, keloid cells showed a reduction in overlap between neighbors (Fig. 7A,B), suggesting that cell-cell adhesion formation in confluent scar fibroblasts is leading to monolayering of the cells. To examine whether enhanced monolayering (*i.e*., a reduction in cellular overlap) upon confluence could explain the increased alignment capacity of keloid fibroblasts, we modeled cellular packing by simulating interactions between cells of similar aspect ratios to real fibroblasts (Fig. 7C, Appendix). Setting a level of cell avoidance based on the observed experimental values of migratory persistence upon confluence led to a similar percentage change in overlap between normal and keloid fibroblasts when comparing experimental and computational results (Supplemental Fig. 4C, Appendix). Unlike previous repulsion-based models that have been used to explain cellular alignment [10], simulating a differential propensity for cellular avoidance did not lead to collision guidance nor alter repulsion dynamics between cells (Supplemental Fig. 3O,P, Appendix). Furthermore, simulations revealed that an increase in cellular avoidance was indeed sufficient to increase alignment (Fig. 7D), suggesting that an enhancement in monolayering behavior upon confluence is leading to a more ordered packing of keloid cells.

**Fig. 8:**
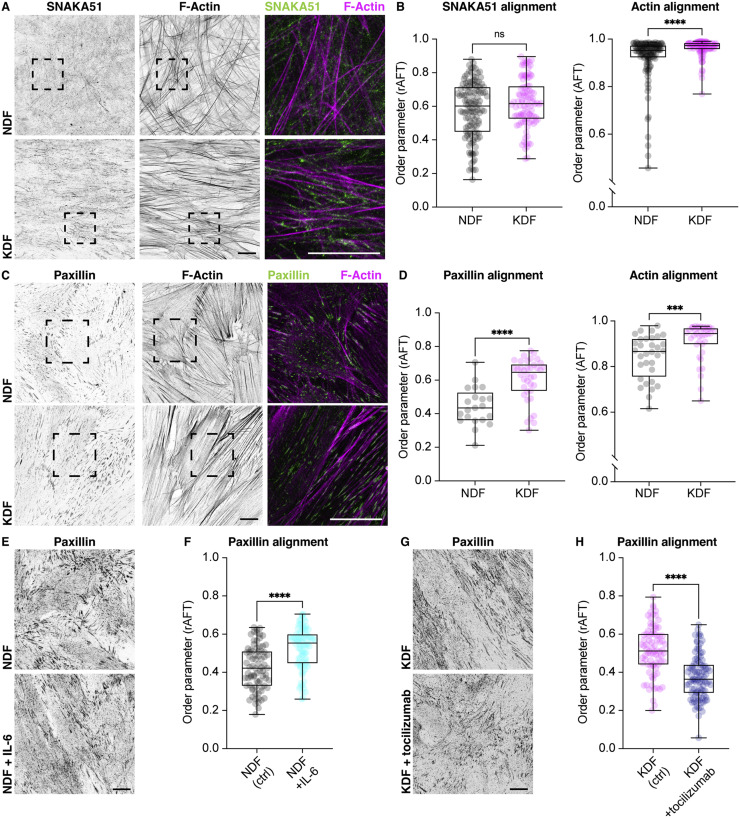
Keloid fibroblasts align focal adhesions upon confluence. **(A)** NDF and KDF stained for F-actin and SNAKA51, a marker of fibrillar adhesions. Note that fibrillar adhesions do not colocalize with actin stress fibers. Right panels are high magnification images of regions highlighted by hatched boxes. **(B)** Quantification of alignment of SNAKA51-labelled adhesions and actin. Despite the increased actin alignment of KDF, no change in alignment of fibrillar adhesions is observed. ns *p* = 0.1785, ****p < 0.0001, Mann–Whitney two-tailed test. Boxplots show medians, 25th and 75th percentiles as box limits, minimum and maximum values as whiskers; each datapoint is displayed as a dot (*n* = 96 NDF, 100 KDF images for SNAKA51, *n* = 124 NDF, 96 KDF images for actin, from 4 biological replicates for each population). **(C)** NDF and KDF stained for mature focal adhesions (paxillin) and F-actin reveal that cell-matrix adhesions are aligned with the actin network in KDF. Right panels are high magnification images of regions highlighted by hatched boxes. **(D)** Quantification of paxillin and actin alignment in (C) reveal that both are aligned. ****p < 0.0001, ***p = 0.0001, Mann–Whitney two-tailed test. Boxplots show medians, 25th and 75th percentiles as box limits, minimum and maximum values as whiskers; each datapoint is displayed as a dot (*n* = 32 NDF, 36 KDF images for paxillin, *n* = 32 NDF, 38 KDF images for actin, from 2 biological replicates for each population). **(E)** NDF with or without IL-6 treatment stained for paxillin. **(F)** Quantification of paxillin alignment in (E) reveals that IL-6 treatment of NDF increases focal adhesion alignment. ****p < 0.0001, Mann–Whitney two-tailed test. Boxplots show medians, 25th and 75th percentiles as box limits, minimum and maximum values as whiskers; each datapoint is displayed as a dot (*n* = 78 NDF ctrl, 84 NDF + IL-6 images from 3 biological replicates for each population). **(G)** KDF with or without tocilizumab treatment stained for paxillin. **(H)** Quantification of paxillin alignment in (G) reveals that tocilizumab (toc) treatment inhibits focal adhesion alignment of KDF. ****p < 0.0001, Mann–Whitney two-tailed test. Boxplots show medians, 25th and 75th percentiles as box limits, minimum and maximum values as whiskers; each datapoint is displayed as a dot (*n* = 61 KDF, 99 KDF + tocilizumab images from 3 biological replicates for each population). Scale bars, 20 µm.

The next question was how this enhanced monolayering in keloid fibroblasts could lead to an aligned ECM. CAF alignment of ECM is correlated with a greater capacity for fibronectin fibrillogenesis [6]. We therefore examined whether *de novo* fibronectin deposition in keloid fibroblasts was distinct since their aligned actin network could be seeding an aligned fibronectin network. However, immunostaining for fibrillar adhesions using an antibody (SNAKA51) specific to active α5β1 integrin [39], which is known to be involved in initial fibronectin assembly, showed no observable difference between keloid and normal cells in terms of fibrillar adhesion alignment (Fig. 8A,B). However, immunostaining for paxillin, a marker of mature focal adhesions, which are thought to be the primary structures responsible for force-transmission to the ECM [40,41], highlighted a highly aligned network of cell-matrix adhesions in keloid fibroblasts (Fig. 8C,D). Additionally, IL-6 treatment of normal fibroblasts increased paxillin alignment, while tocilizumab treatment of keloid fibroblasts decreased paxillin alignment, showing that this response was downstream of IL-6 signaling (Fig. 8E–H). We therefore hypothesize that cell-cell adhesions and supracellular actin network organization may be controlling an alignment of cellular traction forces, which actively remodel and align an underlying ECM network.

### Scar fibroblasts generate an aligned ECM through patterned ECM remodeling

**Fig. 9:**
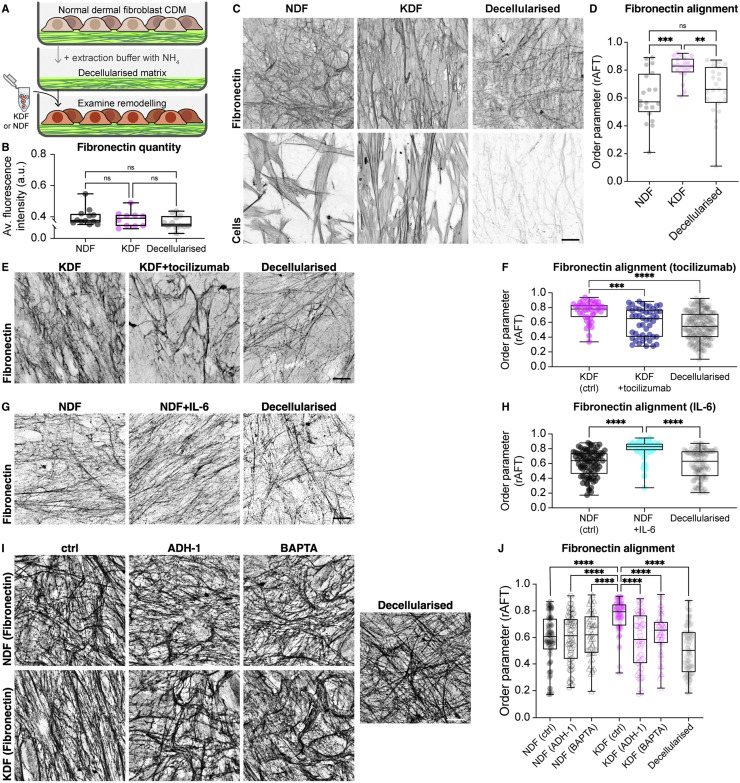
Keloid fibroblasts remodel and align a pre-existing ECM network. **(A)** Schematic showing the experimental strategy to examine the capacity of normal dermal fibroblasts (NDF) and keloid dermal fibroblasts (KDF) to remodel a pre-existing cell derived matrix (CDM). **(B)** Quantification of fluorescence intensity of fibronectin immunostaining after repopulation of NDF and KDF on CDM. There was no statistical difference in fibronectin levels between NDF and KDF or between repopulated CDMs and decellularized matrices revealing that there was little new deposition of ECM. ns *p* > 0.9999 (NDF vs KDF), *p* = 0.2138 (NDF vs Decellularised), *p* = 0.9624 (KDF vs Decellularised), Kruskal-Wallis and Dunn's multiple comparisons test. Boxplots show medians, 25th and 75th percentiles as box limits, minimum and maximum values as whiskers; each datapoint is displayed as a dot (*n* = 12 NDF, 10 KDF, 12 Decellularised images from one biological replicate for each population). **(C)** Fibroblasts plated on top of an isotropic CDM for 72 h and subsequently immunostained for fibronectin highlight that KDF have a capacity to align the pre-existing ECM. Decellularized ECM represents a CDM that has not been repopulated with NDF or KDF. **(D)** Quantification of the data from (C) reveals that KDF have a unique ability to remodel and organize the pre-existing CDM. ***p = 0.0004, **p = 0.0062, ns *p* > 0.9999, Kruskal-Wallis and Dunn's multiple comparisons test. Boxplots show medians, 25th and 75th percentiles as box limits, minimum and maximum values as whiskers; each datapoint is displayed as a dot (*n* = 21 NDF,18 KDF, 22 Decellularised images from one biological replicate for each population). **(E)** KDF with and without tocilizumab treatment plated on top of an isotropic CDM for 72 h and subsequently immunostained for fibronectin. **(F)** Quantification of the data from (E) reveals that tocilizumab treatment of KDF inhibits their capacity to remodel and align the pre-existing CDM. ****p < 0.0001, ***p = 0.0005, ns *p* = 0.4025, Kruskal-Wallis and Dunn's multiple comparisons test. Boxplots show medians, 25th and 75th percentiles as box limits, minimum and maximum values as whiskers; each datapoint is displayed as a dot (*n* = 43 KDF ctrl, 48 KDF + tocilizumab, 155 Decellularised images from 2 biological replicates). **(G)** NDF with and without IL-6 treatment plated on top of an isotropic CDM for 72 h and subsequently immunostained for fibronectin. **(H)** Quantification of the data from (G) reveals that IL-6 treatment of NDF increases their capacity to remodel and align the pre-existing CDM. ****p < 0.0001, ns *p* > 0.9999, Kruskal-Wallis and Dunn's multiple comparisons test. Boxplots show medians, 25th and 75th percentiles as box limits, minimum and maximum values as whiskers; each datapoint is displayed as a dot (*n* = 85 NDF ctrl, 84 NDF + IL-6, 86 Decellularised images from 2 biological replicates). **(I)** Fibronectin immunostaining of CDM after replating of NDF and KDF in the presence or absence of inhibitors of cell-cell adhesion. **(J)** Quantification of ECM remodeling in (I) after replating NDF and KDF in the presence or absence of inhibitors of cell-cell adhesions. KDF uniquely remodel and align the ECM and this capacity is lost in the presence of inhibitors. ****p < 0.0001, Kruskal-Wallis and Dunn's multiple comparisons test. Boxplots show medians, 25th and 75th percentiles as box limits, minimum and maximum values as whiskers; each datapoint is displayed as a dot (*n* = [60 NDF (ctrl)], 59 [NDF (ADH-1)], 47 [NDF (BAPTA)], 59 [KDF (ctrl)], 58 [KDF (ADH-1)], 44 [KDF (BAPTA)], 44 [Decellularised] images from 2 biological replicates). Scale bars, 20 µm.

**Fig. 10:**
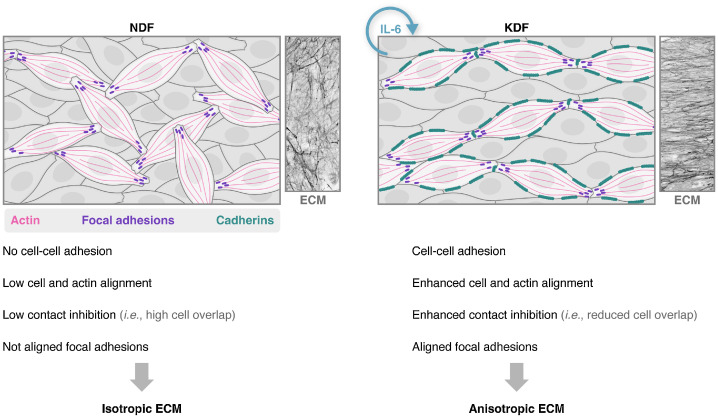
Schematic highlighting the IL-6 mediated mechanism of scar fibroblast ECM anisotropy. Normal dermal fibroblasts (NDF) have few cell-cell adhesions, which results in an increased overlapping between neighboring cells, and low cell/actin/focal adhesion alignment, which leads to a disorganized underlying ECM. In contrast, as a result of autocrine IL-6, keloid dermal fibroblasts (KDF) produce cell-cell adhesions, which helps induce and/or maintain a monolayering-like behavior of the cell population that aids the alignment of the fibroblasts; these cell-cell adhesions also facilitate the nematic organization of a supracellular actin network and alignment of focal adhesions, which leads to an enhanced capacity to produce an aligned ECM.

As CAFs have been reported to physically align a 3D collagen network [6], and keloid fibroblasts have an anisotropic network of cell-matrix adhesions, we examined whether keloid fibroblasts also have a capacity to actively remodel and align a pre-existing ECM network. Normal fibroblasts were used to generate CDMs that are isotropic in nature, and these matrices were then decellularized. We subsequently replated normal and keloid cells onto this isotropic CDM in the absence of ascorbic acid to reduce the new deposition of ECM and examined their capacity to align the pre-existing matrix (Fig. 9A,B). After 72 h, keloid fibroblasts had remodeled the ECM and significantly increased matrix alignment (Fig. 9C,D). Furthermore, treatment of keloid fibroblasts with tocilizumab inhibited the ability to remodel and align the ECM while treatment of normal fibroblasts with IL-6 increased ECM alignment revealing that IL-6 is both necessary and sufficient to induce fibroblast remodeling to align the ECM (Fig. 9E–H). Additionally, inhibition of cell-cell adhesions with BAPTA or ADH-1 prevented keloid remodeling and alignment of the ECM (Fig. 9I,J). Collectively, this demonstrates that keloid fibroblasts, through IL-6-induced intercellular cooperativity, actively remodel the ECM to generate anisotropy in the network (Fig. 10).

## Discussion

Here we show that confluent cultures of keloid fibroblasts generate an anisotropic ECM in culture, characteristic of *in vivo* scar lesions. While TGF-β-dependent myofibroblast differentiation is often assumed to be a major driver of scar formation, data suggest that this is not the upstream factor driving this pathological ECM organization. Rather, cell and ECM alignment is the result of an autocrine IL-6 responsive signaling cascade. In recent years, IL-6 has emerged as a possibly critical mediator of fibrosis in several organ systems (e.g.*,* lung and skin) with inhibitors of the pathway showing clinical promise [42]. IL-6 levels are observed to be increased in fibroblasts from a number of skin pathologies [28,30,42], and a genome-wide association study uncovered a single-nucleotide polymorphism in keloid patients that may lead to hyperactivated signaling in fibroblasts (originally termed the IL-6 amplifier) [43,44]. However, there is little known about the downstream pro-fibrotic functions of IL-6. Work in systemic sclerosis, an autoimmune fibrotic pathology affecting skin and internal organs, revealed that IL-6 inhibition leads to a reduction in fibrotic markers such as α-Smooth Muscle Actin expression and a decrease in dermal thickening, suggesting that IL-6 may indeed have pleiotropic effects on the scarring process [42,45]. Our work indicates that IL-6 leads to an alteration in the architecture of the fibrotic ECM through the modulation of fibroblast behaviors.

It is also intriguing to note that recent evidence has revealed a role for CAF-produced IL-6 in a number of cancers where its expression is correlated with poor prognosis [46], [47], [48], [49], [50]. While the role of IL-6 in cancer development has primarily been hypothesized as paracrine in nature, providing crosstalk between CAFs and cancer cells, it is possible that it may also have an autocrine function in the CAFs themselves to alter their ECM remodeling capacity. Single cell analysis has indicated that only a specific subpopulation of cancer fibroblasts express IL-6, and in pancreatic cancer this IL-6 sub-population was found to be mutually exclusive from α-Smooth Muscle Actin-expressing fibroblasts [47,51], which is consistent with our data suggesting that the cultured keloid fibroblasts are not myofibroblasts. However, CAF expression of IL-6 appears to require paracrine signaling from cancer cells and was reversible when cultured in monolayers [47], unlike keloid fibroblasts, suggesting that keloid cells may be uniquely reprogrammed to maintain high IL-6 expression.

IL-6-dependent fibroblast ECM anisotropy emerges as a result of cellular pre-patterning and alignment of the cells themselves. We hypothesize that the induction of intercellular adhesions between scar fibroblasts leads to several cellular behaviors that may subsequently drive ECM alignment. First, induction of cell-cell adhesion correlates with an enhancement in CIL upon confluence that prevents overlapping between neighboring cells. This subsequently leads to a monolayering behavior and a more ordered packing of the population; simulations suggest that these behaviors may be aiding the initiation or maintenance of the aligned cellular patterns, which in turn drive ECM anisotropy. Additionally, fibroblast cell-cell adhesions lead to formation of a supracellular actin network and alignment of focal adhesions. As cell-cell and cell-matrix adhesions are known to undergo significant mechanochemical crosstalk [52], [53], [54], [55], we speculate that intercellular adhesions lead to the formation of patterned cellular traction stresses, which allow for subsequent remodeling and alignment of the ECM. Indeed, cell-cell adhesions in epithelia generate an imbalance between contacting cells that can alter the pattern of cellular traction forces [53,54]. While keloid fibroblast adhesions are not strong enough to lead to observable collective motion as in epithelial cells, adhesive contacts may be sufficient to align their cell-matrix traction forces that remodel the ECM. We cannot completely rule out a role for *de novo* seeding of an aligned ECM network; however, we do not observe either an alignment of fibrillar adhesions or a difference in contractile behavior, which are both involved in initial fibronectin assembly [22,56]. While fibrillar adhesions do require actomyosin contraction and interact with small actin-microfilaments, they do not colocalize with actin stress fibers [57,58], which are the predominant actin structures that we are measuring in terms of network anisotropy. We therefore speculate that the ECM alignment behavior of keloid fibroblasts is related to a distinct capacity to remodel the ECM.

While fibroblasts are often assumed to only interact sterically with few intercellular adhesions [20,24], there are numerous studies showing that they express cadherins, which lead to functional homotypic and heterotypic cellular interactions [59], [60], [61], [62], [63], [64], [65], [66], [67], [68], [69], [70], [71]. There is also increasing evidence that fibroblast cell-cell adhesions may have active roles during tissue repair and cancer [64,[68], [69], [70],^72^]. Indeed, N-cadherin-mediated collective migration of fibroblasts and their subsequent alignment has recently been observed in models of tissue scarring, and it is interesting to note that this cellular alignment correlated with an aligned ECM [73]. We therefore speculate that intercellular coordination between fibroblasts may also be a general mechanism to remodel the ECM in a patterned fashion in many different activated fibroblast populations, and targeting fibroblast cell-cell adhesions may provide a novel avenue for future therapeutic intervention in tissue fibrosis.

## Data Availability

Data will be made available on request.

## Appendix Supplementary materials

Image, video 1

Image, application 2
