# Supplementary material for: Autocrine IL-6 drives cell and extracellular matrix anisotropy in scar fibroblasts
Source: Matrix Biol. 2023 Nov;123:1–16. doi: 10.1016/j.matbio.2023.08.004 (PMC10878985; doi:10.1016/j.matbio.2023.08.004)
Supplement: Supplementary file 2 [file mmc2.pdf]

Supplementary Figures

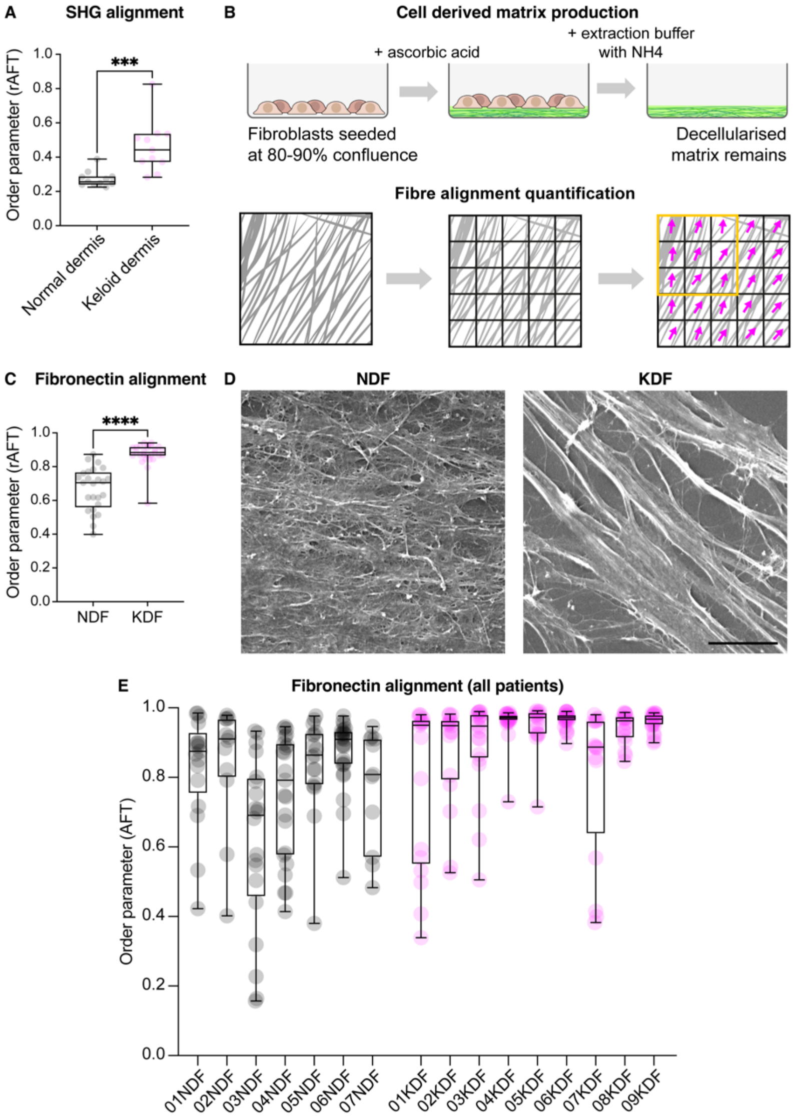

Supplemental Figure 1: Fibroblasts isolated from keloids scars have an enhanced capacity to produce an aligned CDM.

(A) Second Harmonic Generation (SHG) images were acquired from normal and keloid dermis tissue sections to highlight the ECM. The fibers from the SHG signal were subsequently segmented and their alignment quantified, which revealed an increased alignment in keloid scars. ***$p = 0.0002$, Mann–Whitney two-tailed test. Boxplots show medians, 25th and 75th percentiles as box limits, minimum and maximum values as whiskers; each datapoint is displayed as a dot (n = 11 images from one biological replicate for each population). **(B)** Schematic highlighting the quantification of alignment of cell derived matrices (CDM) by AFT (Alignment by Fourier transform). After production and staining of CDM for ECM components, the image is computationally overlayed with a grid and a fast Fourier Transform (FFT) is performed within each window to highlight a predominant fiber direction (represented by the magenta vectors). The order parameter, which is a measure of alignment, is calculated by correlating vector directionality within a user-defined neighborhood of windows (yellow square). While this schematic highlights CDM alignment, the approach can be used to quantify alignment of any fibrillar features. **(C)** Quantification using AFT does not involve feature segmentation. To verify whether results would be similar when first segmenting ECM fibers, we developed a hybrid method (rAFT) where fibers are first segmented by ridge detection and then analysed with AFT. The segmentation approach reveals a similar difference between normal dermal fibroblasts (NDF) and keloid dermal fibroblasts (KDF) alignment as the one obtained when analysing alignment with the FFT alone. ****$p < 0.0001$, Mann–Whitney two-tailed test. Boxplots show medians, 25th and 75th percentiles as box limits, minimum and maximum values as whiskers; each datapoint is displayed as a dot (n = 14 images from one biological replicate for each population). **(D)** Scanning electron microscopy of CDM ultrastructure derived from NDF and KDF samples highlighting an increase in ECM alignment and bundling. Scale bar, 10 µm. **(E)** Quantification of fibronectin alignment in NDF and KDF from multiple patients highlighting the distribution of alignment scores within acquired images. The mean value of these scores for each patient is plotted in Figure 1C. Boxplots show medians, 25th and 75th percentiles as box limits, minimum and maximum values as whiskers; each datapoint is displayed as a dot (n = 12 [01NDF], 32 [02NDF], 25 [03NDF], 15 [04NDF], 20 [05NDF], 23 [06NDF], 11 [07NDF], 17 [01KDF], 16

[02KDF], 27 [03KDF], 17 [04KDF], 26 [05KDF], 18 [06KDF], 22 [07KDF], 20 [08KDF], 16 [09KDF] images from 16 biological replicates).

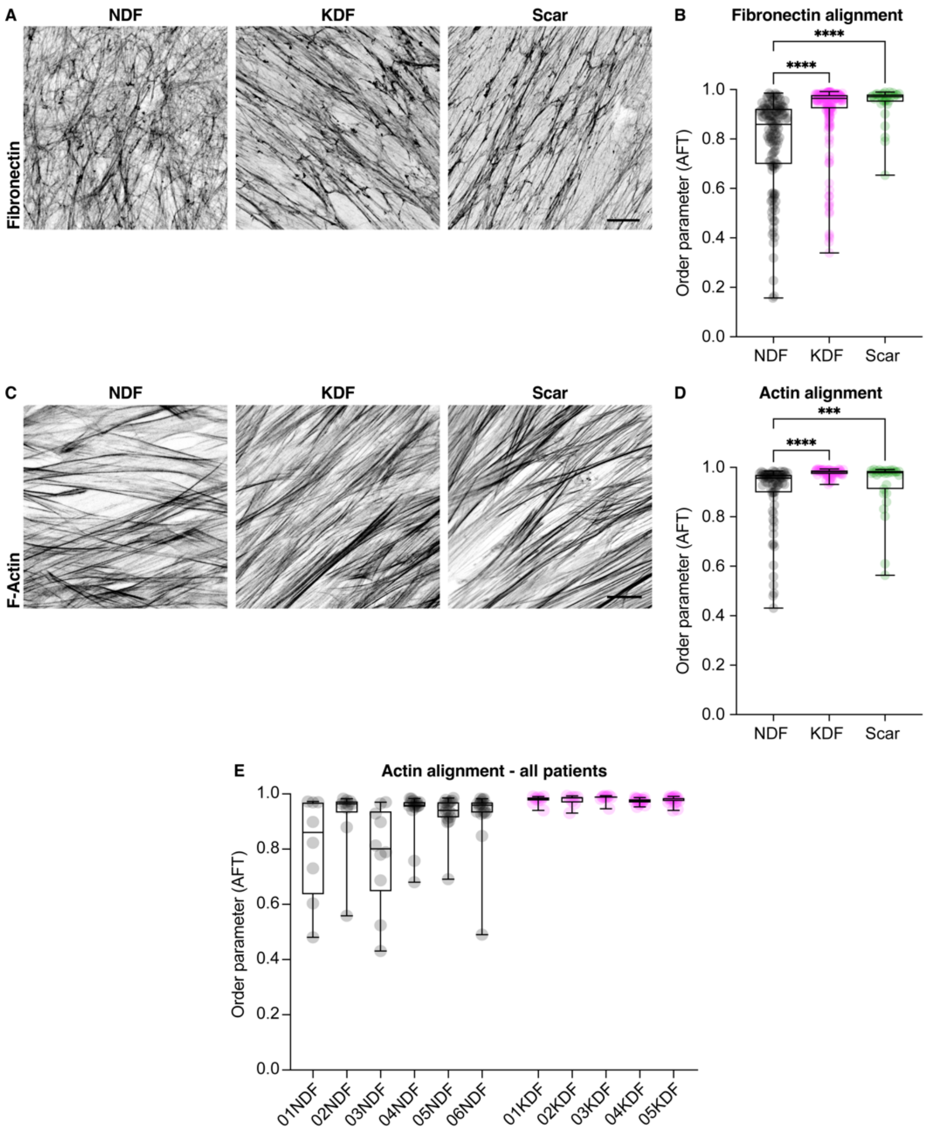
A
NDF
KDF
Scar
Fibronectin
B
Fibronectin alignment
****
****
Order parameter (AFT)
1.0
0.8
0.6
0.4
0.2
0.0
NDF
KDF
Scar
C
NDF
KDF
Scar
F-Actin
D
Actin alignment
***
****
Order parameter (AFT)
1.0
0.8
0.6
0.4
0.2
0.0
NDF
KDF
Scar
E
Actin alignment - all patients
Order parameter (AFT)
1.0
0.8
0.6
0.4
0.2
0.0
01NDF
02NDF
03NDF
04NDF
05NDF
06NDF
01KDF
02KDF
03KDF
04KDF
05KDF

Supplemental Figure 2: Fibroblasts isolated from normal scars have an enhanced capacity to align and produce an aligned CDM.

(A) Fibronectin staining in cell derived matrices (CDM) of normal dermal fibroblasts (NDF), keloid dermal fibroblasts (KDF), and fibroblasts isolated from normal scar tissue. Scale bar, 20 µm. **(B)** Quantification of fibronectin alignment in samples highlighted in (A) reveals increased alignment in both KDF and scar fibroblasts. ****$p < 0.0001$, ns $p = 0.7744$, Kruskal-Wallis and Dunn's multiple comparisons test. Boxplots show medians, 25th and 75th percentiles as box limits, minimum and maximum values as whiskers; each datapoint is displayed as a dot (*n* = 140 NDF images from 8 biological replicates; 179 KDF images from 9 biological replicates; 30 Scar images from 3 biological replicates). **(C)** NDF, KDF, and fibroblasts from normal scars cultured *in vitro* for 5 days and stained for F-actin. Scale bar, 20 µm. **(D)** Quantification of actin alignment from samples highlighted in (C) reveals that KDF and scar fibroblast actin networks are more aligned. ****$p < 0.0001$, ***$p = 0.0006$, ns $p = 0.1141$, Kruskal-Wallis and Dunn's multiple comparisons test. Boxplots show medians, 25th and 75th percentiles as box limits, minimum and maximum values as whiskers; each datapoint is displayed as a dot (*n* = 77 NDF images from 6 biological replicates; 63 KDF images from 5 biological replicates; 30 Scar images from 3 biological replicates). **(E)** Quantification of actin alignment in NDF and KDF from multiple patients highlighting the distribution of alignment scores within acquired images. The mean value of these scores for each patient is plotted in Fig. 2B. Boxplots show medians, 25th and 75th percentiles as box limits, minimum and maximum values as whiskers; each datapoint is displayed as a dot (*n* = 8 [01NDF], 10 [02NDF], 10 [03NDF], 16 [04NDF], 16 [05NDF], 17 [06NDF], 10 [01KDF], 8 [02KDF], 10 [03KDF], 18 [04KDF], 17 [05KDF] images from 11 biological replicates).

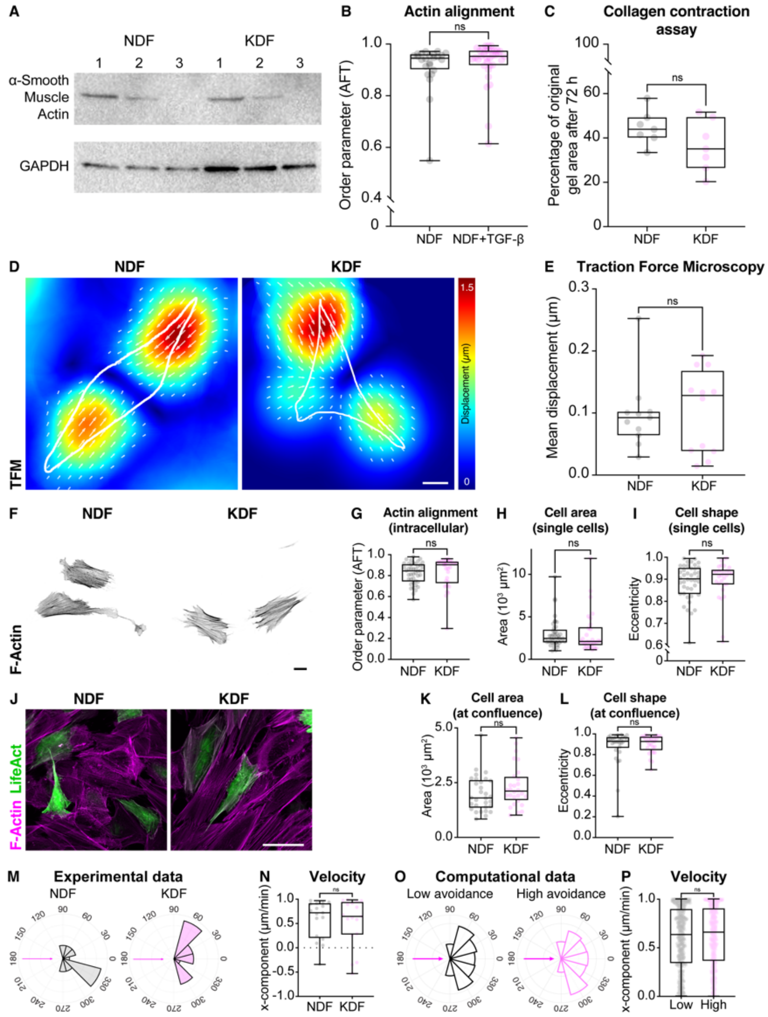

A
NDF
KDF
1 2 3 1 2 3
α-Smooth Muscle Actin
GAPDH
B
Actin alignment
Order parameter (AFT)
NDF
NDF+TGF-β
C
Collagen contraction assay
Percentage of original gel area after 72 h
NDF
KDF
D
NDF
KDF
TFM
Displacement (μm)
E
Traction Force Microscopy
Mean displacement (μm)
NDF
KDF
F
NDF
KDF
F-Actin
G
Actin alignment (intracellular)
Order parameter (AFT)
H
Cell area (single cells)
Area (10³ μm²)
I
Cell shape (single cells)
Eccentricity
J
NDF
KDF
F-Actin LifeAct
K
Cell area (at confluence)
Area (10³ μm²)
L
Cell shape (at confluence)
Eccentricity
M
Experimental data
NDF
KDF
N
Velocity
x-component (μm/min)
O
Computational data
Low avoidance
High avoidance
P
Velocity
x-component (μm/min)
Low
High

Supplemental Figure 3: KDF alignment is unrelated to differences in myofibroblast differentiation, cell contraction, cell morphology, or contact inhibition of locomotion.

(A) Normal dermal fibroblasts (NDF) and keloid dermal fibroblasts (KDF) patient samples cultured *in vitro* were examined for α-smooth muscle actin, a marker of myofibroblast phenotype, by western blot. KDF do not show an increase in myofibroblast differentiation. **(B)** NDF were cultured in the presence or absence of TGF-β1, which induces myofibroblast differentiation. TGF-β1 treatment was insufficient to increase actin alignment. ns $p = 0.8302$, Mann–Whitney two-tailed test. Boxplots show medians, 25th and 75th percentiles as box limits, minimum and maximum values as whiskers; each datapoint is displayed as a dot ($n = 24$ images from one biological replicate for each population). **(C)** NDF and KDF were grown in free-floating polymerized collagen gels and gel contraction was quantified after 72 hours, revealing that KDF do not appear to be more contractile. ns $p = 0.2539$, Mann–Whitney two-tailed test. Boxplots show medians, 25th and 75th percentiles as box limits, minimum and maximum values as whiskers; each datapoint is displayed as a dot ($n = 7$ biological replicates for each population). **(D)** Traction force microscopy (TFM) of NDF and KDF and heatmap showing the magnitude of the substrate displacement. Single cell outline is highlighted in white. Scale bar, 20 μm. **(E)** Quantification of the substrate displacement in (D) reveals no difference between NDF and KDF in their capacity to generate traction forces. ns $p = 0.7399$, Mann–Whitney two-tailed test. Boxplots show medians, 25th and 75th percentiles as box limits, minimum and maximum values as whiskers; each datapoint is displayed as a dot ($n = 11$ NDF, 12 KDF images from 4 biological replicates for each population). **(F)** NDF and KDF cultured at low cell density to analyze actin alignment and cell area in individual cells. Scale bar, 20 μm. **(G)** Quantification of intracellular actin alignment in (F) reveals no difference between NDF and KDF. ns $p = 0.2298$, Mann–Whitney two-tailed test. Boxplots show medians, 25th and 75th percentiles as box limits, minimum and maximum values as whiskers; each datapoint is displayed as a dot ($n = 42$ NDF, 30 KDF cells from one biological replicate for each population). (**H**) Quantification of cell area in individual cells in sparse culture reveals no difference between NDF and KDF, suggesting cells can spread to a similar degree. ns $p = 0.3197$, Mann–Whitney two-tailed test. Boxplots show medians, 25th and 75th

percentiles as box limits, minimum and maximum values as whiskers; each datapoint is displayed as a dot (*n* = 42 NDF, 30 KDF cells from one biological replicate for each population). **(I)** Quantification of cell eccentricity in individual cells in sparse culture reveals no difference between NDF and KDF. ns p = 0.5285, Mann–Whitney two-tailed test. Boxplots show medians, 25th and 75th percentiles as box limits, minimum and maximum values as whiskers; each datapoint is displayed as a dot (*n* = 42 NDF, 30 KDF cells from one biological replicate for each population). **(J)** NDF and KDF plated at high density and stained for F-actin while transfected to mosaically express LifeAct. Scale bar, 50 µm. **(K)** Mosaically labelled NDF and KDF within the confluent monolayers show no difference in cell area. ns p = 0.1747, Mann–Whitney two-tailed test. Boxplots show medians, 25th and 75th percentiles as box limits, minimum and maximum values as whiskers; each datapoint is displayed as a dot (*n* = 30 NDF, 28 KDF cells from 2 biological replicates for each population). **(L)** Mosaically labelled NDF and KDF within the confluent monolayers show no difference in shape eccentricity. ns p = 0.9201, Mann–Whitney two-tailed test. Boxplots show medians, 25th and 75th percentiles as box limits, minimum and maximum values as whiskers; each datapoint is displayed as a dot (*n* = 30 NDF, 28 KDF cells from 2 biological replicates for each population). **(M)** NDF and KDF were cultured at low cell density and individual cell-cell migratory collisions were tracked. Rose plots representing the outgoing velocities of cell collisions with respect to the position of the colliding partner (magenta vector) reveal that both NDF and KDF migrate away from the collisions. **(N)** Quantification of collision dynamics by comparing the x-components of the outgoing velocity unit vectors reveals no statistical difference between KDF and NDF. ns p > 0.9999, Mann–Whitney two-tailed test. Boxplots show medians, 25th and 75th percentiles as box limits, minimum and maximum values as whiskers; each datapoint is displayed as a dot (*n* = 17 colliding cells from one biological replicate for each population). **(O)** Simulated collision events using parameters for cell avoidance that match experimental results and simulations assessing cellular alignment (Figure 7C,D, Appendix). Rose plots representing the outgoing velocities of cell collisions with respect to the position of the colliding partner (magenta vector) reveal that altering cell overlap avoidance has no effect on collision dynamics. This result is comparable to experimental collision data in panel M. **(P)** Quantification of simulated collision dynamics by comparing the x-components of the outgoing velocity unit vectors reveals no statistical difference

between low and high cell overlap avoidance. This result is comparable to experimental collision data in panel N. ns $p = 0.4028$, Mann–Whitney two-tailed test. Boxplots show medians, 25th and 75th percentiles as box limits, minimum and maximum values as whiskers; each datapoint is displayed as a dot ($n = 600$ simulated colliding cells).

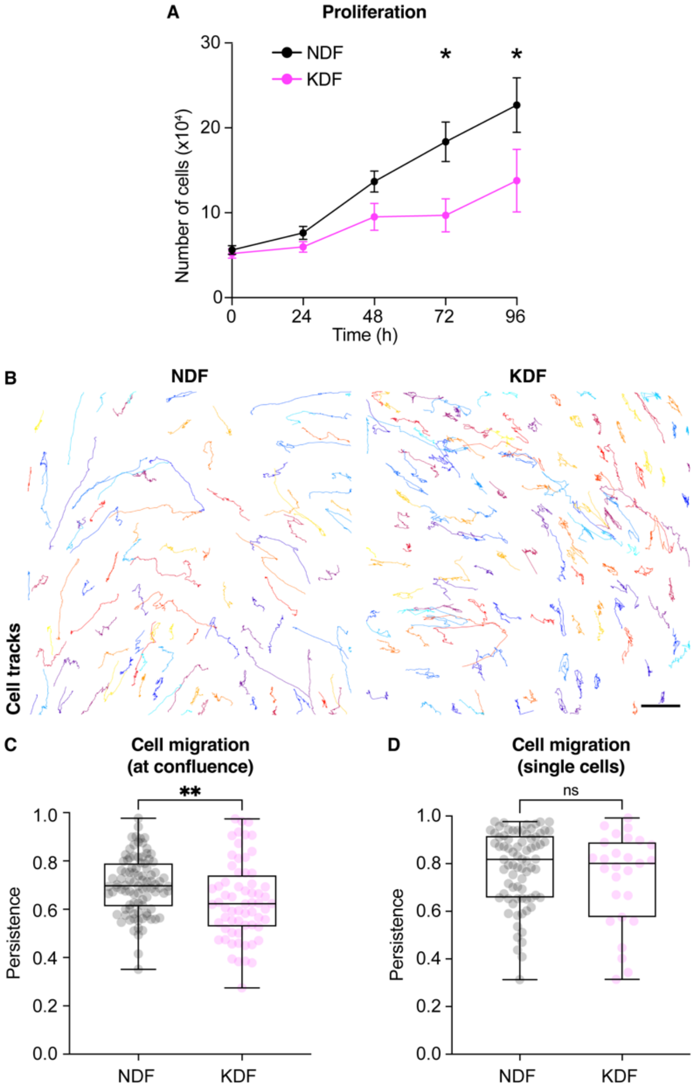
A
Proliferation
NDF
KDF
Number of cells (x10⁴)
Time (h)
0
24
48
72
96
0
10
20
30
*
*
B
NDF
KDF
Cell tracks
C
Cell migration
(at confluence)
**
Persistence
NDF
KDF
D
Cell migration
(single cells)
ns
Persistence
NDF
KDF

Supplemental Figure 4: Keloid fibroblast monolayering behavior is correlated with a change in contact inhibition upon reaching confluence.

(A) Normal dermal fibroblasts (NDF) and keloid dermal fibroblasts (KDF) were quantified over time in culture, which revealed that KDF are ultimately slower to proliferate. ns $p > 0.9999$ (0h), $p = 0.9830$ (24h), $p = 0.5377$ (48h); *$p = 0.0156$ (72h), $p = 0.0123$ (96h), repeated measures two-way ANOVA and Šidák's multiple comparisons test. Dots represent mean values and bars represent the standard error of the mean for each time point (n = 6 biological replicates for each population). **(B)** NDF and KDF migration was tracked in confluent cultures. Scale bar, 50 µm. **(C)** Quantification of the track persistence in confluent cultures as in (B) reveals a reduction in persistence in KDF. **$p = 0.0022$, Mann–Whitney two-tailed test. Boxplots show medians, 25th and 75th percentiles as box limits, minimum and maximum values as whiskers; each datapoint is displayed as a dot (n = 94 NDF, 64 KDF cells from one biological replicate for each population). **(D)** Quantification of the persistence of individual cell migration in low density cultures shows no difference between NDF and KDF, revealing that the decrease in persistence in (C) is specifically related to cells at confluence. ns $p = 0.3684$, Mann–Whitney two-tailed test. Boxplots show medians, 25th and 75th percentiles as box limits, minimum and maximum values as whiskers; each datapoint is displayed as a dot (n = 76 NDF, 27 KDF cells from one biological replicate for each population).

Video 1: Live imaging of dense cell cultures reveals a monolayering-like behavior of keloid fibroblasts.

Normal (NDF) and keloid (KDF) fibroblast cultures were plated at high density and live imaged overnight. Nuclear tracking (magenta) reveals that keloid cells show a reduced propensity to migrate over each other, which leads to a decrease in their persistence and a monolayering-like behavior. Timestamp = Hours:Minutes

Experimental procedures

Tissue collection, section preparation and analysis

The collection of normal skin, keloid scar tissue and normal scar tissue from patients providing informed consent was ethically approved by the National Research Ethics Service (UK) (14/NS/1073). The study was conducted in accordance with the ethical standards as set out in the WMA Declaration of Helsinki and the Department of Health and Human Services Belmont Report. Over the course of the project, 15 each of normal and keloid skin tissue samples and 3 normal scar tissue samples were analyzed.

Unfixed tissue samples were embedded in optimal cutting temperature compound (OCT, Life Technologies) prior to sectioning. Fresh frozen tissue sections were sectioned at 12 µm using a Thermo Cryostar Nx70 (Thermo Fisher Scientific) and fixed using 4% paraformaldehyde (PFA) for 20 minutes at room temperature. For immunohistochemistry, sections were blocked using a phosphate buffered saline (PBS) buffer containing 5% goat serum and 0.3% Triton-X 100 for 1 hour at room temperature. They were then incubated at 4°C overnight +/- primary antibody diluted in antibody dilution buffer (1% BSA containing 0.3% Triton-X 100). Following overnight incubation, sections were well rinsed in PBS and incubated with phalloidin 488 (dilution 1:500; Life Technologies), DAPI (0.5 µg/ml; Sigma) and fluorescent secondary antibody Alexa Fluor 568 (1:500; Life Technologies) in antibody dilution buffer for 1 hour at room temperature. Tissue sections were rinsed in PBS and diH_2O and mounted using DAKO mounting media. Actin and DAPI images were acquired using a Zeiss LSM 880 equipped with a 63x Plan-Apochromat oil objective (NA 1.4). For second harmonic generation imaging, tissue sections were imaged using a Zeiss LSM 7MP equipped with a 20x water immersion objective (NA 1.0).

Cell isolation and culture

Aside from the patient matched NDF and KDF sample, keloid tissue was collected using a surgical procedure that does not remove skin from the normal margins and fibroblasts were isolated from the entire scar region. Primary dermal cells were isolated from de-epithelialized skin (removed using Dispase II (1 U/ml in Hank's Balanced Salt Solution, Sigma) overnight at 4°C) either by enzymatic digestion

(Whole Skin Dissociation Kit, Miltenyi Biotec) or by *ex vivo* explant culture. Fibroblasts were maintained at 37°C and 5% CO_2 in high-glucose DMEM supplemented with 10% FBS (Hyclone), 1% Penicillin-Streptomycin (Sigma), 1% pyruvate (Sigma) and 4 mM L-Glutamine (Sigma).

Fibroblasts were treated with either ADH-1 (100 μM; Exherin, Cambridge Bioscience), BAPTA (200 μM; Abcam) or DMSO (1:1000) either at the time of plating on gelatin coated coverslips and fixed at 24 hours or following 24 hours on gelatin coated coverslips and fixed 48 hours later. Normal dermal fibroblasts were treated with IL-6 (10 ng/ml) or control of 0.0001% BSA (bovine serum albumin in PBS) at the time of plating or at 24 hours after plating on coverslips. Keloid dermal fibroblasts were treated with the IL-6 receptor inhibitor tocilizumab (50 μg/ml; MedChemExpress) or Human IgG1 isotype control (1 μg/ml; BioLegend) either at time of cell seeding or at 24 hours after cell seeding.

Collagen gel contraction assay

Collagen gel contraction was assessed by mixing 500 μl of fibroblast suspension at 1.5 x 10^5 cells/ml, 200 μl of ~3mg/ml rat tail collagen I solution and 6 μl of 1M NaOH. The mixture was added to a well of a 24 well plate and left for ~15 minutes at room temperature to allow polymerization. Following the addition of complete culture medium to the well, the collagen gel was then released from the edge of the well in order to examine contraction potential in free-floating gels. Collagen gel area was imaged at 72 hours using the BioRad Gel Doc+ imaging system and contraction quantified using ImageJ.

Cell number quantification

For proliferation analysis, equal numbers (5x10^4) of fibroblasts were seeded in triplicate wells of a 24-well plate then counted using a hemocytometer daily over 4 days.

Cell derived matrix (CDM) preparation

CDMs were prepared according to a previously published protocol [1, 2]. Briefly, cleaned glass coverslips (13 mm diameter, VWR) were incubated for 1 hour with

sterile 0.2% (wt/vol) gelatin (Sigma) at 37°C. Coverslips were then rinsed and the gelatin crosslinked with 1% glutaraldehyde (Alfa Aesar) for 30 minutes at room temperature. Coverslips were rinsed with sterile PBS and incubated with 1 M Glycine (Sigma) for 20 minutes at room temperature to quench crosslinking. Coverslips were rinsed with PBS and incubated with complete media for 30 minutes at 37°C and either used immediately or stored at 4°C. Fibroblasts were seeded at near confluency and left overnight to adhere and spread. Once they formed a confluent monolayer, fibroblasts were treated with ascorbic acid (Sigma) at 50 µg/ml every other day for 5 days. To decellularize CDMs, coverslips were incubated at 37°C with a solution of 10 mM ammonium hydroxide and 0.25% Triton X-100 in PBS. Once decellularization was complete, matrices were washed with PBS before fixation with 4% paraformaldehyde (PFA) or further processing.

To examine the role of IL-6 in matrix deposition, CDMs were produced using KDF treated with the IL-6 receptor inhibitor tocilizumab (50 µg/ml; MedChemExpress) or Human IgG1 isotype control (1 µg/ml; BioLegend) at time of cell seeding and at 24 hours after plating alongside ascorbic acid addition. NDF CDMs were created by treating normal fibroblasts with IL-6 (10 ng/ml) or vehicle control (0.0001% BSA) at the time of plating and at 24 hours after cell seeding along with ascorbic acid treatment. All samples were decellularized and fixed on day 3.

For CDM remodeling experiments, following decellularization the matrices were subsequently incubated with 10 µg/ml DNase (Roche) in sterile PBS at 37°C for 30 minutes. The CDMs were then well rinsed with sterile PBS and either used immediately or stored at 4°C until cells were ready to be plated. Prior to the matrices being repopulated with either normal or keloid fibroblasts, matrices were incubated for 1 hour at 37°C with complete medium.

Immunofluorescence

For immunofluorescence of cellular samples, samples were fixed in 4% PFA and stored in PBS prior to staining. Cells were permeabilized using 0.2% Triton-X 100 (Sigma) for 10 minutes at room temperature before being well rinsed with PBS. Following permeabilization, samples were blocked for 1 hour at room temperature

using 4% bovine serum albumin (BSA) in PBS. To allow visualization of adhesions, cells were incubated with primary antibodies to paxillin (1:500, Abcam), β-catenin (1:500; Cell Signaling Technologies) or N-cadherin (1:250; Cell Signaling Technologies) for 1 hour at room temperature or integrin α5 (1:500, Sigma) overnight at 4°C. Cells including CDMs were incubated overnight at 4°C with anti-fibronectin (1:500; Abcam) in 4% BSA. Following incubation with primary antibodies, samples were well rinsed in PBS and incubated with fluorescent secondary antibody (1:500; Life Technologies), phalloidin (1:500 dilution; Life Technologies) and DAPI (0.5 μg/ml; Sigma) in 4% BSA for 1 hour at room temperature. Finally, samples were rinsed and mounted using Dako mounting medium (Agilent Technologies). Analysis of the actin alignment following treatments was carried out by staining only for F-actin using phalloidin and DAPI as above.

To examine α -catenin localisation, cells were fixed at 72 hours following plating using 4% PFA containing 0.1% Triton-X 100 for 15 minutes. Cells were then permeabilized with 0.25% Triton-X 100 for 5 minutes and then washed with PBS containing 0.1% Tween (PBS-T, Sigma). Cells were then blocked in PBS-T containing 10% goat serum for 1 hour prior to incubation with anti α-catenin antibody (1:500; Sigma) for 1 hour at room temperature. Following incubation with primary antibody, samples were rinsed with PBS-T and incubated with fluorescent secondary antibody (1:500; Life Technologies), phalloidin (1:500 dilution; Life Technologies) and DAPI (0.5 μg/ml; Sigma) in PBS-T containing 10% goat serum for 1 hour at room temperature before being rinsed in PBS and H_2O and mounted using Dako mounting medium.

Fixed samples were imaged using a Zeiss LSM 880 equipped with a 40x NA 1.3 Plan-Apochromat oil objective, 40x NA 1.1 LD C-Apochromat water objective, or 63x NA 1.4 Plan-Apochromat oil objective.

To compare cell alignment and actin alignment, fibroblasts were seeded onto 35 mm dishes (Ibidi) and fixed at 24 hours. They were stained using phalloidin and DAPI as above. Following staining, samples were stored in PBS at 4°C until imaged using a Zeiss LSM 880 10x NA 0.30 air objective.

Decellularized CDM samples fixed on coverslips were blocked using 4% BSA in PBS for 1 hour at room temperature and then incubated overnight at 4°C with anti-fibronectin (1:500; Abcam) and/or anti-type I collagen (1:2000; Abcam) primary antibodies in 4% BSA. Following overnight incubation, samples were rinsed with PBS and incubated with fluorescent secondary antibodies Alexa Fluor 488 (1:500; Life Technologies) and Alexa Fluor 568 (1:500; Life Technologies) in 4% BSA before being rinsed and mounted using Dako mounting medium. Decellularized matrices were imaged using a Zeiss LSM 880 equipped with 63x NA 1.4 Plan-Apochromat oil.

LifeAct transfection

Cell shape and cell spread area were examined using confluent monolayer cultures of fibroblasts plated onto gelatin coated coverslips. Mosaic expression of EGFP-LifeAct was carried out by transfecting fibroblasts with EGFP-Lifeact using jetPRIME (Polyplus) transfection reagent. Media was changed 4 hours after transfections and fibroblasts were fixed after 48 hours using 4% PFA, with F-actin and nuclei visualized as described above.

Time-lapse microscopy

For cell tracking experiments, fibroblasts were seeded either sparsely or at confluency and left to adhere and spread overnight on a 24 well plate (Ibidi). SiR-DNA (Spirochrome) was added (0.5 μM/ml) at least 1 hour prior to imaging and images were acquired every 10 minutes for at least 12 hours using a 20x 0.8 NA objective.

Scanning Electron Microscopy

Samples were fixed for 1 hour with 2.5% glutaraldehyde in 0.1 M cacodylate buffer (pH 7.3) at room temperature. After the initial fixation, samples were rinsed several times in cacodylate buffer and placed in 0.1% Tannic acid in water for 30 minutes. Samples were then washed and dehydrated in a graded series of ethanol and critical point dried using a Leica CPD300 (Leica Microsystems). Dried samples were mounted on stubs with adhesive carbon tabs (TAAB Laboratories) and sputter coated with gold (4 nm) using a Leica ACE600 (Leica microsystems Vienna) before

examination by scanning electron microscopy using a JEOL JCM7000 Neoscope (JEOL Japan) operated at 15 kV in high vacuum mode. Signals were collected using the secondary electron detector.

Isolation of cell conditioned media

Keloid and normal fibroblasts were seeded in T75 flasks at a density of $1x10^6$ cells/flask with high-glucose DMEM supplemented with 10% FBS, 1% Penicillin-Streptomycin, 1% pyruvate and 4 mM L-Glutamine. Keloid and normal conditioned media were collected after 48 hours and centrifuged at 1300 rpm for 5 minutes. Collected media were either immediately used to treat cells or stored at -80 ^{o}C.

IL-6 quantification

Quantification of the level of IL-6 expression in fibroblasts isolated from normal and keloid dermis was carried out by Human DuoSet ELISA (R&D Systems) in high binding 96 well plates. The plate was prepared as per the manufacturer's protocol with minor modifications. Fibroblasts were plated in complete medium, and supernatant collected at 24 hours. 100 µL of standards or samples (neat and 1:10 diluted in reagent diluent) were added to the plate which was sealed and incubated for 2 hours at room temperature. The plate was subsequently rinsed 3 times using wash buffer (0.05% Tween in PBS). Following this, 100 µL of the detection antibody diluted in reagent diluent was added to the plate and the plate was covered and incubated for 2 hours at room temperature followed by 3 rinses with wash buffer. The plate was then incubated with 100 µL of the working dilution of Streptavidin-HRP for 20 minutes at room temperature. This was followed by 3 rinses with wash buffer before 100 µL of TMB substrate (1:1 solution of Reagents A&B from kit) was added and the plate incubated for 5-20 minutes before the stop solution of 50 µL of 0.16M sulfuric acid was added. The optical density of the plate was measured at 420 nm on a spectrophotometer plate reader.

Overlap

Cell overlap was visualised by labelling fibroblasts with CellTrace reagents. Confluent fibroblasts were incubated for 15 minutes at 37 ^{o}C with CellTrace Violet (2 µM in PBS) or CellTrace CFSE (5 µM in PBS). Following this the CellTrace solution was removed, the cells were rinsed 3 times before being incubated for 10 minutes at

37 °C with complete medium. The cells were then trypsinised and seeded in 35 mm dishes (Ibidi) at a ratio of 9:1 CellTrace Violet cells:CellTrace CFSE cells and fixed with 4% PFA 24 hours after plating. Cell overlap was imaged using a Zeiss LSM 880 equipped with 20x NA 0.8 Plan-Apochromat air objective.

Traction force microscopy

Hydroxy-polyacrylamide hydrogels containing 0.2 µm fluorescent beads (FluoSpheres Carboxylate-Modified Microspheres 505/515, Thermo Fisher Scientific) with a Young's Modulus of 10 kPa were prepared following a previously described protocol [3]. Briefly, solution A was prepared with 500 µL of 40% acrylamide (Bio-Rad) and 65 µL of hydroxyethyl-acrylamide (Sigma Aldrich). 500 µL of solution A was mixed with 250 µL of 2% bis-acrylamide (Bio-Rad) to form solution B. For a 10 kPa gel, 150 µL of solution B was thoroughly mixed in 340 µL of PBS and 10 µL of fluorescent beads. Gels were polymerised with 5 µL 10% APS (Sigma Aldrich) and 1.5 µL TEMED (Sigma Aldrich) on 35mm dishes (Ibidi). For cell seeding, gelatin was conjugated to the surface of the hydrogel using the bifunctional cross-linker Sulfo-SANPAH (Thermo Fisher Scientific) as detailed in [4]. Gels were sterilised for 10 minutes under UV in tissue culture hood prior to cell seeding. Cells were incubated with SPY555-actin (Spirochrome) and SPY650-DNA (Spirochrome) 2 hours prior to live imaging. Cells and fluorescent beads were imaged on a Zeiss LSM 880 equipped with a 20x 0.8 NA objective fitted with a stage incubator (37°C and 5% CO_2). Cells were removed from the gel using 10X trypsin (Thermo Fisher Scientific).

In order to quantify traction forces, maximum intensity projection of fluorescent beads pre- and post-trypsin treatment were collated to a stack and registered using a slice alignment plugin, StackReg, in Fiji to correct for experimental drift [5]. Bead displacement was quantified by PIV as described in [6] (https://github.com/stemarcotti/PIV), with the following parameters: source size 4 µm, search size 8 µm, grid distance 2 µm, correlation threshold 0.5. The vector field was interpolated with a Gaussian kernel (size 40 µm, sigma 10 µm) and the mean displacement calculated for each image.

Western blot

Cells were lysed directly into radioimmunoprecipitation assay (RIPA) buffer. Equal quantities of protein were subjected to SDS-PAGE and western blotting using the Invitrogen NuPAGE system as recommended by the manufacturer (Life Technologies). The PVDF membrane was blocked in 5% skim milk powder in tris buffered saline with 0.1% Tween-20 (TBS-T) for at least 1 hour before incubation with the primary α-smooth muscle actin (αSMA) antibody (Abcam) or GAPDH antibody (Abcam) overnight at 4°C. The subsequent day, the membrane was washed 3x 10 minutes in TBS-T prior to incubation with HRP-conjugated anti-rabbit secondary antibody (Jackson Laboratories) for 2 hours at room temperature. After 3x 10 minutes additional washing in TBS-T, the membrane was subjected to chemiluminescence (SuperSignal West Pico Plus, Thermo Scientific) and visualized using a BioRad Gel Doc+ imaging System.

Quantification of fiber anisotropy

Image pre-processing. Images were pre-processed in Fiji to highlight the fibrillar structures. To this aim, the contrast was enhanced (0.35% saturated pixels) and subsequently a local contrast adjustment was performed ("CLAHE" plugin, default parameters). To quantify fiber anisotropy, we used two different approaches depending on the characteristics of the images, AFT (Alignment by Fourier Transform) or rAFT (ridge detection + AFT), as detailed below.

AFT (Alignment by Fourier Transform). The first methodology relies on a 2D Fast Fourier Transform (FFT) representation of the image as previously described [7, 8] and is available as an open-source package (AFT, https://github.com/OakesLab/AFT-Alignment_by_Fourier_Transform). Briefly, each image is subdivided into smaller overlapping windows; for each window, the corresponding FFT is calculated. A predominant fiber orientation yields an asymmetrically skewed FFT, with the direction of the asymmetry related to the orientation of the fibers. This calculation results in quantifying a local orientation (*i.e.,* angle) in each window in the form of a vector field encompassing the entire image.
The size of the window was set to 10 μm (50% overlap) for all images; exceptions were made for data acquired at lower magnification to account for lower resolution of

the fibrillar features (window size of 20 μm), and when looking at intracellular actin in single cells to obtain details at the required length scale (window size of 5 μm).
To correlate the alignment of the ECM and actin, the cosine squared of the local orientation difference between the two vector fields was computed, showing values close to one for similar angles.
To assess global alignment, an order parameter was computed on the obtained vector field within each 5 by 5 neighborhood of windows. The median value across all neighborhoods within an image was then calculated, with values close to 1 indicating highly aligned fields of view. The size of such neighborhood corresponds to an area representing ~3-4 cell diameters, where an optimal difference between the NDF and KDF populations was observed in actin alignment. Order parameters calculated over increasingly larger neighborhoods were used to evaluate the length scale of the observed anisotropy.
All calculations were performed in MATLAB (Mathworks, v. R2018b). This approach was used in all instances in which the experimental images showed a relatively continuous field of fibrillar structures (either fibronectin or actin). Additionally, this FFT-based quantification is much faster computationally than segmentation-based methods, which allowed for analysis of larger sample sizes and experimental perturbations. In all other cases where there was a significant presence of gaps in the signal, a second methodology that relied on fiber segmentation was employed.

rAFT (ridge detection + AFT). This second methodology is based on a similar approach to the one used by TWOMBLI [9] and was used to quantify fibrillar feature alignment whenever the signal was discontinuous, as the grid-based FFT approach creates spurious vectors when features are absent (namely, second harmonic generation imaging, collagen and focal adhesion immunostaining, and CDM remodeling). First, a ridge detection step is performed in Fiji [10] to segment the fibrillar features (the following parameters were used: line width = 20, low contrast = 0, high contrast = 120, minimum branch length = 10). Then, the segmented image is run through AFT as detailed above.

Quantification of nuclear alignment

Images were pre-processed in Fiji to segment the nuclei. To this aim, an automatic thresholding was performed, followed by removal of particles smaller than 10 pixels

(“Analyse particles” plugin) and filling of holes within the mask. The processed images were imported in MATLAB, where custom-written routines were used to quantify alignment. The orientation of each nucleus was obtained with the *regionprops* function and the order parameter [8] was calculated for the nuclei directors across the whole image or across a local neighborhood of 100 µm x 100 µm for images acquired at a lower magnification.

Single cell area and shape

Single cells or mosaically expressed cells within a confluent monolayer were segmented by thresholding in Fiji. The masks of segmented cells were imported in MATLAB, where the *regionprops* function was used to measure their area and eccentricity. The eccentricity is measured as the ratio of the distance between the foci and the major axis length of an ellipse fitted to each cell; ellipses whose eccentricity is zero or one degenerate in a circle or in a line segment, respectively.

Quantification of cell layering

Mosaically expressed single cells (sparse channel) and the confluent monolayer they sit within (confluent channel) were segmented in Fiji. This resulted in binary masks where pixels assigned a value of one represent regions covered by cells in both the sparse and confluent channels for each image. Binary masks of single cells (sparse channel) were then split with the function *Find connected regions* to obtain masks only containing one cell. Clusters of cells or cells on the edge of the field of view were discarded. Each of the obtained single cell masks was then compared against the corresponding confluent channel binary mask in MATLAB. A percentage overlap was calculated as the number of pixels with a value of one in both channels over the total number of pixels in the single cell mask.

Collision dynamics

Contact inhibition of locomotion (CIL) of fibroblasts was analyzed as previously described [11]. The position of two colliding cells was recorded 120 min before collision, at the time of collision, and 120 min after collision. A spatial normalization was performed to align the colliding cell to its colliding partner at the time of collision on a horizontal director. Outgoing velocity unit vectors were then computed in

MATLAB and their horizontal component (i.e., x component) used to compare the dynamics of the NDF and KDF populations.

Migration persistence

The persistence index was calculated as the ratio between the shortest possible distance travelled and the actual distance traversed by each cell in a 3-hour interval. To this aim, each cell track was divided in intervals of such length by a walking windowing method, and the persistence index for each interval was calculated and averaged to obtain a single value per track. Values close to one indicate high persistence, with the cell moving on an ideal straight line between the start and end point.

Statistics

Statistical tests were performed in Prism (GraphPad, v. 9). Mann-Whitney two-tailed tests or Kruskal-Wallis tests were used when comparing two or more than two samples, respectively. Dunn's multiple comparisons test was employed to compare the mean rank of each sample with the mean rank of every other sample after Kruskal-Wallis statistical significance was observed. For the proliferation assay, a repeated measures two-way ANOVA followed by a Šidák's multiple comparisons test was used. Significance is reported in figure panels as follows: "****" for p-values lower than 0.0001, "***" lower than 0.001, "**" lower than 0.01, "*" lower than 0.05, "ns" or not stated otherwise.

The alignment decay was fitted with linear least squares regression, with the y-intercept constrained to one. High fitting quality was obtained in all cases, with R^2 scores higher than 0.95.

References

[1] G. Theocharidis, Z. Drymoussi, A.P. Kao, A.H. Barber, D.A. Lee, K.M. Braun, J.T. Connelly, Type VI Collagen Regulates Dermal Matrix Assembly and Fibroblast Motility, J Invest Dermatol 136(1) (2016) 74-83.
[2] J. Franco-Barraza, D.A. Beacham, M.D. Amatangelo, E. Cukierman, Preparation of Extracellular Matrices Produced by Cultured and Primary Fibroblasts, Curr Protoc Cell Biol 71 (2016) 10 9 1-10 9 34.
[3] J. Rheinlaender, A. Dimitracopoulos, B. Wallmeyer, N.M. Kronenberg, K.J. Chalut, M.C. Gather, T. Betz, G. Charras, K. Franze, Cortical cell stiffness is independent of substrate mechanics, Nat Mater 19(9) (2020) 1019-1025.
[4] S.V. Plotnikov, B. Sabass, U.S. Schwarz, C.M. Waterman, High-resolution traction force microscopy, Methods Cell Biol 123 (2014) 367-94.
[5] P. Thevenaz, U.E. Ruttimann, M. Unser, A pyramid approach to subpixel registration based on intensity, IEEE Trans Image Process 7(1) (1998) 27-41.
[6] L. Yolland, M. Burki, S. Marcotti, A. Luchici, F.N. Kenny, J.R. Davis, E. Serna-Morales, J. Muller, M. Sixt, A. Davidson, W. Wood, L.J. Schumacher, R.G. Endres, M. Miodownik, B.M. Stramer, Persistent and polarized global actin flow is essential for directionality during cell migration, Nat Cell Biol 21(11) (2019) 1370-1381.
[7] M. Cetera, G.R. Ramirez-San Juan, P.W. Oakes, L. Lewellyn, M.J. Fairchild, G. Tanentzapf, M.L. Gardel, S. Horne-Badovinac, Epithelial rotation promotes the global alignment of contractile actin bundles during Drosophila egg chamber elongation, Nat Commun 5 (2014) 5511.
[8] S. Marcotti, D. Belo de Freitas, L.D. Troughton, F.N. Kenny, T.J. Shaw, B.M. Stramer, P.W. Oakes, A Workflow for Rapid Unbiased Quantification of Fibrillar Feature Alignment in Biological Images, Frontiers in Computer Science 3(91) (2021).
[9] E. Wershof, D. Park, D.J. Barry, R.P. Jenkins, A. Rullan, A. Wilkins, K. Schlegelmilch, I. Roxanis, K.I. Anderson, P.A. Bates, E. Sahai, A FIJI macro for quantifying pattern in extracellular matrix, Life Sci Alliance 4(3) (2021).
[10] C. Steger, An unbiased detector of curvilinear structures, Ieee T Pattern Anal 20(2) (1998) 113-125.
[11] J.R. Davis, C.Y. Huang, J. Zanet, S. Harrison, E. Rosten, S. Cox, D.Y. Soong, G.A. Dunn, B.M. Stramer, Emergence of embryonic pattern through contact inhibition of locomotion, Development 139(24) (2012) 4555-60.

Appendix to: Autocrine IL-6 drives cell and extracellular matrix anisotropy in scar fibroblasts

Fiona N. Kenny, Stefania Marcotti, Deandra Belo De Freitas, Elena Drudi, Vivienne Leech, Rachel E. Bell, Jennifer Easton, Maria-del-Carmen Diaz-de-la-Loza, Roland Fleck, Leanne Allison, Christina Philippeos, Angelika Manhart, Tanya Shaw, Brian Stramer

1 Mathematical model of fibroblast alignment

Modelling Strategy. We build a mechanistic agent based model to describe the motion of individual fibroblasts interacting with neighbouring cells via cell overlap avoidance. Unlike the fibroblast models presented e.g. in [Li et al., 2017] and [Wershof et al., 2019] we don't model interactions with the ECM. To test the hypothesis that the experimentally observed difference in cell alignment between KDFs and NDFs is based on differences in cell overlap avoidance, we derive a force based model, where cells are modeled as "soft" bodies in the sense that overlap is allowed, but punished by a force potential. The model shares characteristics with the model [Li et al., 2017], however we use a different cell shape and force potential.

Model Derivation. We know that fibroblasts are very dynamic in shape but are observed to be roughly spindle shaped. We therefore model individual fibroblasts as ellipses. We consider N fibroblasts within our fixed domain $\Omega \in \mathbb{R}^2$, each with position $\mathbf{X}_i = (X_i, Y_i) \in \mathbb{R}^2, i = 1, \ldots, N$ and orientation $\alpha_i \in [0, 2\pi), i = 1, \ldots, N$. Each fibroblast is described by an ellipse with semi-major axis a and semi-minor axis b as shown in Fig 1A. In the absence of other cells, fibroblasts self-propel with constant velocity v_0 in direction $\mathbf{e}(\alpha_i) := (\sin(\alpha_i), \cos(\alpha_i))^T$, subject to orientational noise modeled by independent Brownian motions dB_t^i of intensity w_n. When two fibroblasts come into contact with each other and overlap, they try to minimise this overlap, causing each cell to experience an equal and opposite repulsion force that affects their position,

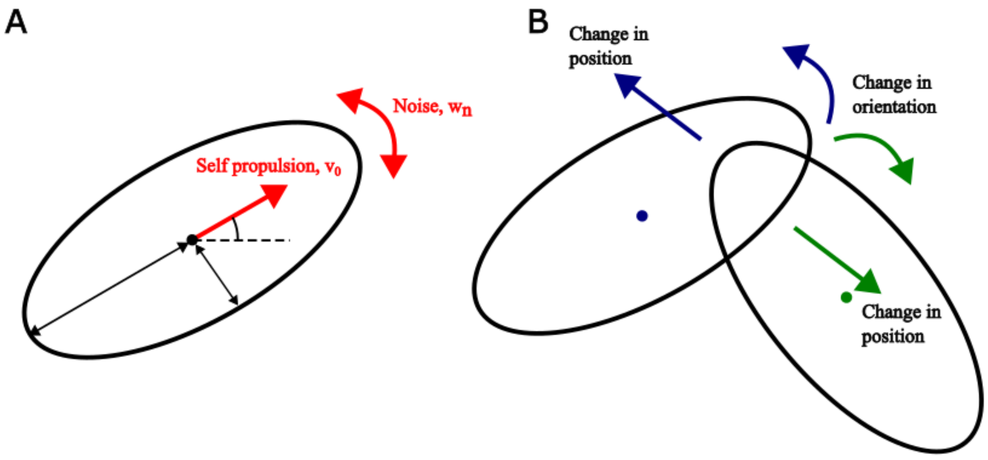

Figure 1: A: Schematic explaining cell dimensions a and b, cell position (X_i, Y_i), orientation α_i, self propulsion v_0 and noise w_n. B: Schematic of effect of overlap avoidance on position and orientation.

and a torque that affects their orientation as shown in Fig 1B.

We derive the governing equations using energy minimisation. We parameterise the points inside a cell positioned at $\mathbf{X}(t)$ with orientation $\alpha(t)$ at time t by

$$\mathbf{r}(t,s,\theta) = \mathbf{X}(t) + s\mathbf{R}(\alpha(t))\mathbf{v}(\theta), \qquad s \in [0,1],\ \theta \in [0,2\pi),$$

where the rotation matrix $\mathbf{R}(\alpha)$ and the vector $\mathbf{v}(\theta)$ are defined by

$$\mathbf{R}(\alpha) = \begin{pmatrix} \cos(\alpha) & -\sin(\alpha) \\ \sin(\alpha) & \cos(\alpha) \end{pmatrix} \qquad \text{and} \qquad \mathbf{v}(\theta) = \begin{pmatrix} a\cos(\theta) \\ b\sin(\theta) \end{pmatrix}.$$

For a small, positive time step Δt, we then define an energy potential, $E_{\Delta t}$ of the form

$$E_{\Delta t} = \int_0^{2\pi} \int_0^1 \left[\eta \frac{|\mathbf{r}(t,s,\theta) - \mathbf{r}(t+\Delta t, s, \theta)|^2}{2\Delta t} + V(\mathbf{r}(t,s,\theta)) \right] abs\, \mathrm{d}s\, \mathrm{d}\theta,$$

where the first term models friction with the environment with friction parameter η, and the second corresponds to a force potential V that will incorporate overlap avoidance. The factor abs is the area element and is a consequence of the chosen parameterisation.

We obtain governing equations by minimising this energy potential. To derive equations for $\mathbf{X}$ and α, we differentiate with respect to $\mathbf{X}$ and α respectively (treating all other variables in the energy potential as constants). We then set the derivative to zero and take the limit $\Delta t \to 0$. Together with the self-propulsion and the orientational noise we then obtain the following stochastic differential equations for the motion of one cell

$$\begin{aligned} \mathrm{d}\mathbf{X} &= -\frac{\mathrm{d}t}{\eta\pi} \int_0^{2\pi} \int_0^1 s \nabla V \, \mathrm{d}s\, \mathrm{d}\theta + v_0 \mathbf{e}(\alpha) \mathrm{d}t, \\ \mathrm{d}\alpha &= -\frac{4\mathrm{d}t}{\eta\pi(a^2+b^2)} \int_0^{2\pi} \int_0^1 \nabla V \cdot s^2 \left(\mathbf{R}\mathbf{v}(\theta)\right)^{\perp} \, \mathrm{d}s\, \mathrm{d}\theta + \sqrt{2w_n}\, \mathrm{d}B_t^i. \end{aligned}$$

The superscript $\perp$ describes the left-turned normal vector. For two overlapping ellipses with domains A and B, we let our force potential be the function $V(\mathbf{r}) = \tilde{\sigma} \mathbb{1}_{A\cap B}(\mathbf{r})$ where $\mathbb{1}_{A\cap B}(\mathbf{r})$ is the indicator function which equals 1 if $\mathbf{r} \in A \cap B$ and 0 otherwise. The strength of this potential is $\tilde{\sigma} \in \mathbb{R}$. If $\tilde{\sigma} > 0$, the cells experience repulsion in response to overlap, and if $\tilde{\sigma} < 0$, the cells experience attraction. In this work $\tilde{\sigma} > 0$. Two cells only experience overlap avoidance upon overlapping with each other, hence we define $\mathcal{N}_i$ as the set of indices of cells that overlap with the i-th cell.

Final Model. Using V as defined above, we can re-write our governing equations such that they depend only on the points of overlap between cells i and j, denoted by $\mathbf{Y}_k^{ij}$, where up to $k = 4$ points of overlap are possible. In the following $K_{ij} = 1$ or $K_{ij} = 2$ denotes the number of overlap point pairs between cell i and cell j (ignoring borderline cases).

$$\mathrm{d}\mathbf{X}_i = -\frac{\tilde{\sigma}}{\eta ab\pi} \mathbf{R}(\alpha) \sum_{j\in\mathcal{N}_i} \sum_{k=1}^{K_{ij}} (\mathbf{Y}_{2k-1}^{ij} - \mathbf{Y}_{2k}^{ij})^{\perp} \mathrm{d}t + v_0 \mathbf{e}(\alpha)\, \mathrm{d}t, \tag{1}$$

$$\mathrm{d}\alpha_i = \frac{2\tilde{\sigma}}{\eta ab\pi(a^2+b^2)} \sum_{j\in\mathcal{N}_i} \sum_{k=1}^{K_{ij}} (|\mathbf{X_i} - \mathbf{Y}_{2k}^{ij}|^2 - |\mathbf{X_i} - \mathbf{Y}_{2k-1}^{ij}|^2)\, \mathrm{d}t + \sqrt{2w_n}\, \mathrm{d}B_t^i.$$

From (1) we see that cells' centers are being pushed in the direction normal to vector connecting the points of overlap. Note that the parameters $\tilde{\sigma}$ and η only appear as the ratio $\tilde{\sigma}/\eta$, but not individually, i.e. only

the ratio of the strength of overlap avoidance and friction matter. In the following we therefore define $\sigma = \tilde{\sigma}/\eta$, but will refer to σ as the strength of overlap avoidance, since we assume the friction to be constant. These two governing equations are supplemented with initial conditions and boundary conditions. We use randomly distributed orientations and random initial positions with a condition imposed on the minimum distance between cells (30 μm) so they do not start with too much overlap. We work on a square domain $\Omega = [0, L]^2$ and use periodic boundary conditions.

2 Parameters and Quantifiers

Parameter choices. Some parameters of the model can be drawn directly from the experimental data: We obtain the values of $a = 41.15\mu$m, $b = 17.94\mu$m from fitting ellipses to experimentally determined cell shapes, and $v_0 = 0.47\mu$m/min from experimentally determined cell speed. Further we choose $L = 600\mu$m as a representative domain size, $N = 145$ as the typical number of cells in that domain and the final time $T = 750$ minutes as the maximal simulation time (by which dynamics seem to have reached a steady state). We fix the amount of noise to a relatively low value of $w_n = 0.0015$/min based on the observation that too much noise seems to destroy the alignment dynamics. Below we explain how the remaining parameter σ (the ratio between overlap avoidance and friction) is inferred from the data. Tab. 1 lists the relevant parameters and their values.

Simulation quantifiers. We use the same quantifiers as for experimental results, namely persistence, alignment and overlap. They are determined analogously as for the experimental measurements (see main paper): The persistence is the ratio of the shortest distance travelled by a cell from start to finish, divided by its total track length. This is calculated as a walking average over 3 hours of acquisition with one time frame every 10 minutes. Cells that leave the domain in this time are discounted. The alignment (order) parameter used to compute the alignment over different neighbourhood sizes is

$$Q = 2(\langle \cos^2(\theta_{ij}) \rangle - \frac{1}{2}),$$

where θ_{ij} is the difference in angle between a randomly chosen reference cell i and cell j within a circular neighbourhood of diameter given by the neighbourhood size. $\langle \cdot \rangle$ denotes the average over all cells. The process of choosing a reference cell and then calculating the alignment based on its neighbours is repeated until all pairs of cells have been considered with no repeats. Further details of the alignment parameter can be found in [Marcotti et al., 2021]. Cell overlap is calculated by identifying the percentage of each cell area

Name	Meaning	Value	Comment
a	Semi-major axis	41.15 μm	Fitted from data
b	Semi-minor axis	17.94 μm	Fitted from data
v_0	Self-propulsion speed	0.47μm/min	Fitted from data
w_n	Noise parameter	0.0015/min	Estimated
$\sigma = \frac{\tilde{\sigma}}{\eta}$	Ratio between overlap avoidance and friction	NDF: 90 μm^2/min KDF: 130 μm^{2}min	Fitted using experimental values of persistence
L	Size of domain	600 μm	Similar to experimental data.
N	Number of cells in the domain	145	Similar to experimental data

Table 1: List of parameters.

that is overlapping with neighbouring cells and then averaging over all cells in the domain. We note that for our simulations below the overlap is much smaller than experimentally determined. This is likely due the simplified representation of cell shape. Since there are two sources of randomness (initial conditions and orientational noise), we always average quantifiers over 50 simulations.

3 Simulation results

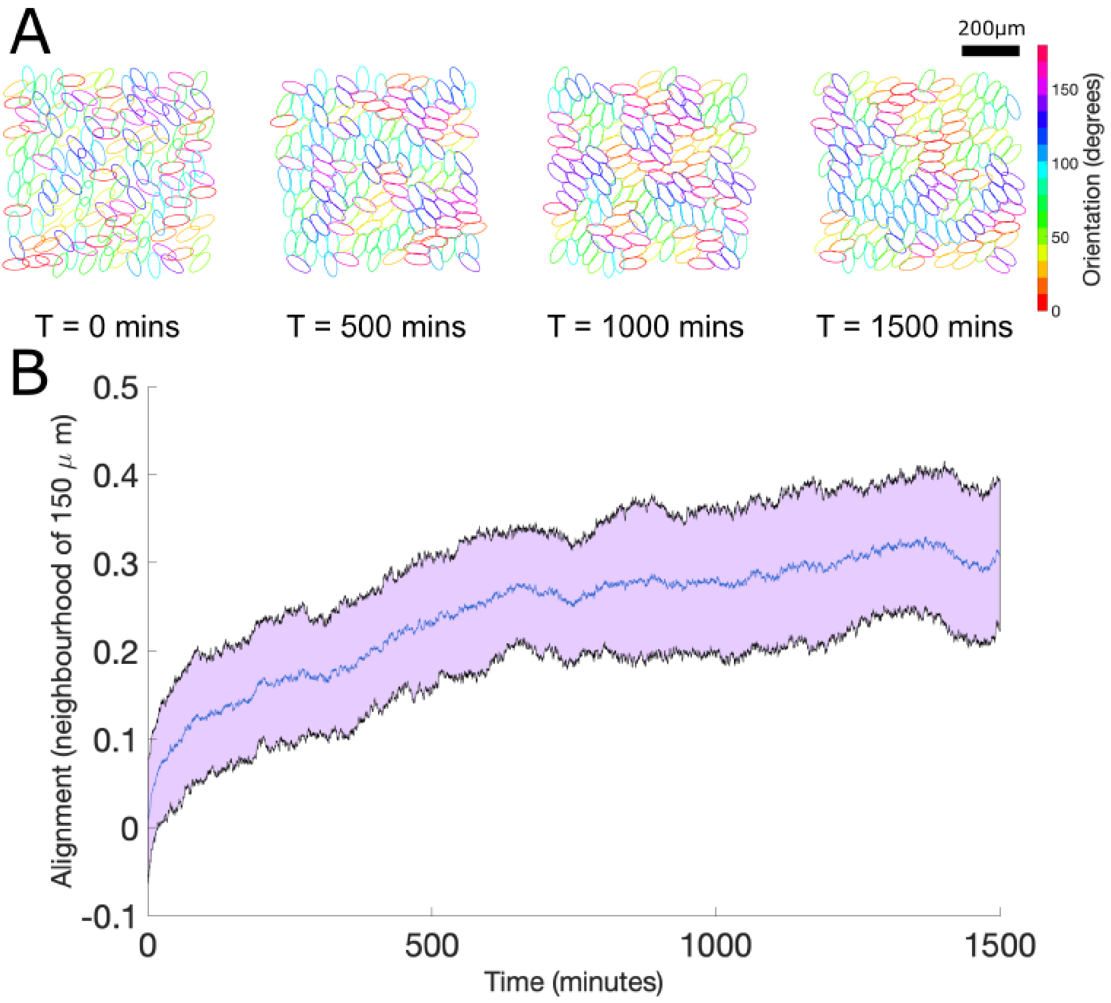

Figure 2: A: Simulation snapshots of an example simulation at different time point. Colours reflect orientation. B: Alignment calculated over a neighbourhood size of 150μm for simulations with parameters as in A. Shown is the mean (blue line) ± standard deviation (shaded) of 50 simulations.

Simulation method. To simulate the governing equations we use a standard first order forward Euler method. The computationally expensive part of the simulation is identifying points of overlap between ellipses. To compute this we define 200 points on the edge of each ellipse that are equally spaced by angle. To identify points of overlap we search for points on the edges of nearby ellipses which are within a certain optimal range. This gives a computationally more efficient, approximate method to find points of overlap.

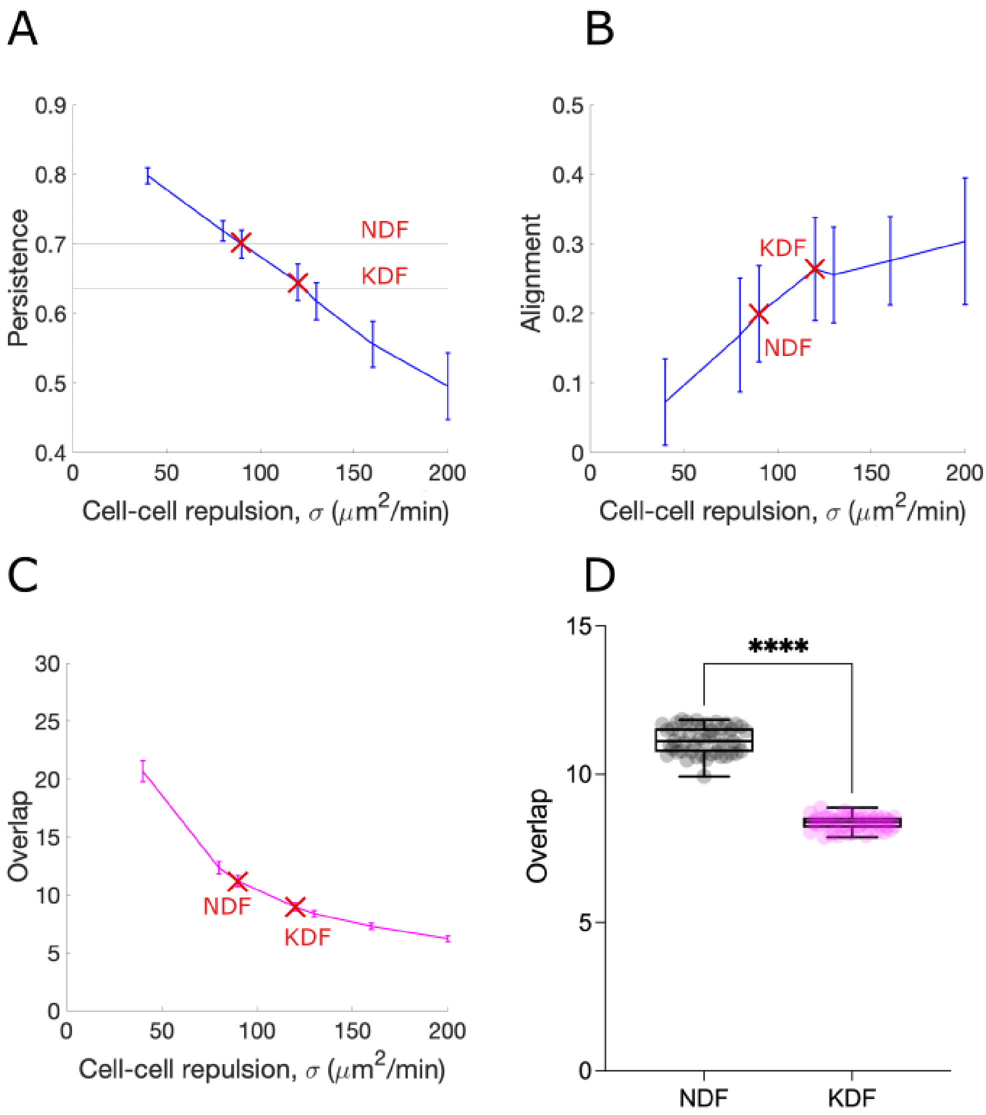

Figure 3: A-C: Persistence, alignment and overlap calculated for different values of cell overlap avoidance strength at 750min. Shown are mean values and standard deviation for 50 simulation runs. A: Horizontal lines mark experimentally determined values of persistence for normal (NDF) and keloid fibroblasts (KDF) at high confluence. Red crosses mark the consequently chosen values for σ. Values of σ corresponding to NDF and KDF are marked in red crosses. D: Boxplot showing the cell overlap results for the NDF and KDF parameter sets. Each datapoint is displayed as a dot.

Typical alignment dynamcis. In Fig. 2A we see snapshots of an example simulation with parameters $a = 41.15\mu\text{m}$, $b = 17.94\mu\text{m}$, $w_n = 0.0015/\text{min}$, $\sigma = 130\frac{\mu\text{m}^2}{\text{min}}$. We see that the cells start with a random orientation and then form pockets of alignment as time progresses. Fig. 2B shows the typical time evolution for the averaged alignment parameter for the example simulation. We see that the alignment parameter starts at around zero when the cells are randomly oriented and then increases as time goes on until it starts to plateau off at a value of around 0.3.

Influence of overlap avoidance. Next we want to investigate the effect of σ, which determines the strength of overlap avoidance (compared to the substrate friction). We find that more overlap avoidance leads to less persistent cells, more alignment and less cell overlap, as seen in Fig. 3A, B, C. We know that the persistence of both cell types of cells is similar at low confluence, and becomes different between the two types when the fibroblasts are at high confluence. This suggests that we can fix the amount of noise in the system and vary the overlap avoidance to obtain different values for persistence at confluence. From Fig. 3A we can see that this leads to the keloid fibroblasts having a higher strength of overlap avoidance than the normal fibroblasts. This in turn leads to less overlap and more alignment which qualitatively corresponds with what is seen experimentally. In Fig. 3D we see the resulting difference in overlap. While the absolute overlap values are lower than those obtained experimentally (see discussion above), the percentage difference in these values quantitatively matches with experimental values.

Quantifying collision dynamics. To compare to experimental collision measurements and asses if differences in collision dynamics are predicted to be evident in individual cell collisions, we performed a computational collision essay, analogous to the experimental collision essay shown in Supp.Fig 4M,N. For this we use the fitted parameters from above and run simulations containing only two cells. We initialise so that cells collide head to head at 15 relative orientations between 0 and 2π. We record the cells' orientations 120 minutes after the collision and perform the same normalisation as done experimentally and described in the Material and Methods. To account for the noise in the system, we repeat each collision 20 times and then average over the results. As shown in Supp.Fig 4O,P the results match the experimental values, in particular no difference in behaviour between NDFs and KDFs can be seen.

References

[Li et al., 2017] Li, X., Balagam, R., He, T.-F., Lee, P. P., Igoshin, O. A., and Levine, H. (2017). On the mechanism of long-range orientational order of fibroblasts. *Proceedings of the National Academy of Sciences - PNAS*, 114(34):8974–8979.

[Marcotti et al., 2021] Marcotti, S., Belo de Freitas, D., Troughton, L. D., Kenny, F. N., Shaw, T. J., Stramer, B. M., and Oakes, P. W. (2021). A workflow for rapid unbiased quantification of fibrillar feature alignment in biological images. *Frontiers in Computer Science*, 3.

[Wershof et al., 2019] Wershof, E., Park, D., Jenkins, R. P., Barry, D. J., Sahai, E., and Bates, P. A. (2019). Matrix feedback enables diverse higher-order patterning of the extracellular matrix. *PLoS computational biology*, 15(10):e1007251–e1007251.
